# Indicators to evaluate quality of care in head and neck cancer in Spain

**DOI:** 10.1007/s12094-023-03298-z

**Published:** 2023-10-17

**Authors:** Juan Jesús Cruz Hernández, Virginia Arrazubi Arrula, Yolanda Escobar Álvarez, Almudena García Castaño, Juan José Grau de Castro, Lara Iglesias Docampo, Julio Lambea Sorrosal, Pedro Pérez Segura, Antonio Rueda Domínguez, Francisco J. Campos-Lucas, Irene Santamaría Rodríguez, Maria Bessa, Paula Gratal, Fernando Caballero-Martínez, Diana Monge Martín, Cristina Antón-Rodríguez, Rafael López

**Affiliations:** 1 Departamento de Medicina, Universidad de Salamanca, Consejero Emérito de la Fundación ECO, Campus Universitario Miguel de Unamuno s/n, 37007 Salamanca, Spain; 2 Fundación ECO, Madrid, Spain; 3 Servicio de Oncología Médica, Hospital Universitario Basurto, Bilbao, Spain; 4 Servicio de Oncología Médica, Hospital General Universitario Gregorio Marañón, Madrid, Spain; 5 Servicio de Oncología Médica, Hospital Universitario Marqués de Valdecilla, Santander, Spain; 6 Servicio de Oncología Médica, Hospital Clínic de Barcelona, Barcelona, Spain; 7 Servicio de Oncología Médica, Hospital 12 de Octubre, Madrid, Spain; 8 Servicio de Oncología Médica, Hospital Clínico Universitario Lozano Blesa, Saragossa, Spain; 9 Servicio de Oncología Médica, Hospital Clínico San Carlos, Madrid, Spain; 10 Servicio de Oncología Médica, Hospital Regional Universitario de Málaga, Málaga, Spain; 11 Facultad de Medicina, Universidad Francisco de Vitoria, Madrid, Spain; 12 Servicio de Oncología Médica, Hospital Clínico Universitario e Instituto de Investigación Sanitaria (IDIS) de Santiago de Compostela, CIBERONC, Santiago de Compostela, Spain

**Keywords:** Head and neck cancer, Delphi, Quality of care, Criteria, Indicators, Spain

## Abstract

**Purpose:**

This study aimed to develop a set of criteria and indicators to evaluate the quality of care of patients with head and neck cancer (HNC).

**Methods:**

A systematic literature review was conducted to identify valuable criteria/indicators for the assessment of the quality of care in HNC. With the aid of a technical group, a scientific committee of oncologists specialised in HNC used selected criteria to propose indicators that were evaluated with a two-round Delphi method. Indicators on which consensus was achieved were then prioritised by the scientific committee to develop a final set of indicators.

**Results:**

We proposed a list of 50 indicators used in the literature or developed by us to be evaluated with a Delphi method. There was consensus on the appropriateness of 47 indicators in the first round; the remaining 3 achieved consensus in the second round. The 50 indicators were scored to prioritise them, leading to a final selection of 29 indicators related to structure (3), process (22), or outcome (4) and covering diagnosis, treatment, follow-up, and health outcomes in patients with HNC. Easy-to-use index cards were developed for each indicator, with their criterion, definition, formula for use in real-world clinical practice, rationale, and acceptable level of attainment.

**Conclusions:**

We have developed a set of 29 evidence-based and expert-supported indicators for evaluating the quality of care in HNC, covering diagnosis, treatment, follow-up, and health outcomes.

**Supplementary Information:**

The online version contains supplementary material available at 10.1007/s12094-023-03298-z.

## Introduction

Head and neck cancers (HNC) comprise a broad spectrum of tumours arising in the oral cavity, nasopharynx, paranasal sinus cavity, oropharynx, larynx, and hypopharynx [1]. HNC represents approximately 5% of all cancers (excluding non-melanoma skin cancers) and 5% of all cancer deaths [1]. Most HNC are squamous cell carcinomas (HNSCC) and are diagnosed at advanced stages [2]. Management of patients with HNC is complicated and requires highly specialised multidisciplinary care [2]. The therapeutic approach varies with stage and tumour location. Overall, surgery or radiotherapy are the treatments of choice for early locoregional disease; locally advanced disease is treated with either surgery plus (chemo)radiotherapy or chemoradiotherapy alone [3, 4]. Patients with metastatic and/or recurrent disease are often not amenable to surgery or curative radiotherapy [5] and are treated instead with systemic therapy, including immunotherapy [3, 4]. Integration of immunotherapy in the management of recurrent and/or metastatic disease has considerably altered the management of HNC, and combinatorial approaches are being studied [5].

Quality of care affects outcomes of patients with HNC [6], and highly variable quality of care for these patients has been reported in hospitals across Europe and the USA [6–8]. Radiotherapy, in particular, is one of the main factors that impact patient outcomes [9–12]. In an international phase III trial, good compliance with radiotherapy protocols led to the best results; the quality of radiotherapy also highly correlated with the number of patients enrolled at each centre [12]. Moreover, expertise in radiotherapy appears to play a role in the outcomes of patients with HNC, who experienced improved survival when treated at high-volume centres [10] or at centres that enrolled a larger number of patients into clinical trials [11, 12]. These findings highlight the importance of specialisation and quality in the adequate management of HNC.

Currently, there are no widely accepted or recommended criteria or indicators for evaluating the quality of care in HNC. The Quality Oncology Practice Initiative (QOPI^®^), developed by the American Society of Clinical Oncology (ASCO), includes a set of core measures for evaluating the quality of care in any cancer, as well as some cancer-specific measures [13]. However, QOPI^®^ currently has no HNC-specific measures [13]. Quality of care indicators for HNC developed by other working groups or institutions are limited in their scope, as they focus primarily on diagnosis and follow-up, include few treatment-related measures, and lack indicators of structure [14, 15]. Quality assurance initiatives in HNC focus on particular steps of patient management but do not encompass the complete patient journey from diagnosis to follow-up. One of these initiatives included a patient panel for more comprehensive development of measures, but the indicators are listed rather than fully defined and have not been updated since 2016 [14]. The national healthcare system of Scotland developed 15 well-defined indicators; however, specific disease stages were not considered, and a multidisciplinary group of specialists was not involved in their development [15].

In Spain, there are no guidelines recommending specific criteria and indicators for evaluating the quality of care in HNC. Fundación ECO (Excellence and Quality in Oncology)—a Spanish foundation of senior oncologists from the main Spanish hospitals involved in cancer treatment—developed this study in collaboration with Universidad Francisco de Vitoria. Here, we present a comprehensive expert- and evidence-based set of indicators driven by the healthcare community for evaluation of the quality of care in HNC, with detailed instructions on how to use the indicators in clinical practice.

## Methods

### Study design

**Fig. 1:**
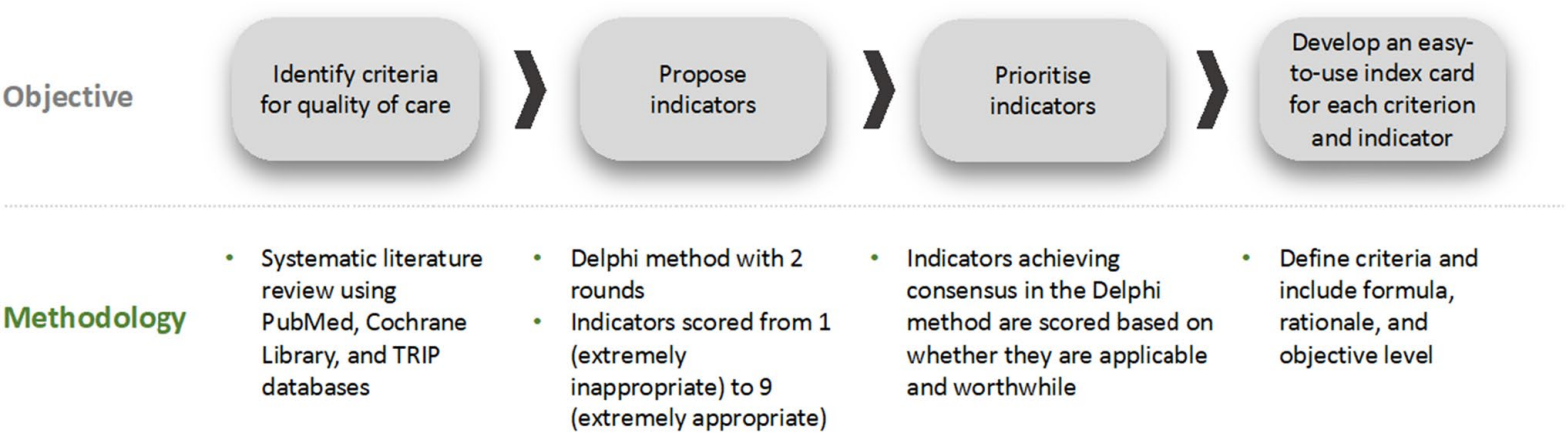
Study design

The project was developed over 4 stages (Fig. 1): (1) Systematic literature review and selection of criteria and indicators; (2) Two-step modified Delphi method evaluating the appropriateness of the indicators; (3) Prioritisation of indicators; (4) Final development of indicators and standards. Three groups of participants were involved in the project: a scientific committee, a technical group from Universidad Francisco de Vitoria (experts in the Delphi method and quality of healthcare), and a Delphi panel comprising clinical experts in HNC.

### Scientific committee

Nine oncologists who are experts in HNC participated in every stage of this study until the development of the index cards with indicators to implement in clinical practice. After committee recruitment was completed, the topic, methodology, and goals of the consensus process were presented to the panel in a virtual meeting.

### Systematic literature review

The literature review was conducted following Cochrane guidelines (with the exception of having only one reviewer) to identify criteria and indicators of interest related to screening, diagnosis, treatment, and follow-up of HNC. Search parameters focused on HNC, guidelines, quality indicators, performance indicators and evaluation of outcomes. Relevant documents published in English or Spanish were identified by searching in PubMed, Cochrane Library and Trip databases, as well as in institutional websites for scientific associations and national health services. Literature published between 2015 and 2020 was prioritised; the review was completed in September 2020.

### Delphi consensus method

Criteria and indicators of interest were extracted from the selected literature and evaluated by the scientific committee, who developed additional ones. The appropriateness of the proposed measures was evaluated with a two-round Delphi method that took place between March and April 2021 via web-based surveys. Delphi respondents scored the indicators on a scale of 1 (extremely inappropriate) to 9 (extremely appropriate). The results from the first survey were shared by email with the scientific committee. The indicators on which consensus was not achieved were asked again in the second round with no modifications. After the second round, the results were discussed in a virtual meeting.

### Development of measures and standards

After the Delphi method was completed, all indicators on which consensus was reached were further evaluated to identify those most important for implementation in clinical practice. The scientific committee scored the appropriateness of indicators, taking into account whether they were applicable (use of resources, workload) and worthwhile (adequate effort–benefit of implementing them). Those with the highest mean score were selected for the final list of recommendations.

The technical group developed index cards for each indicator, following the model used by the Spanish Society of Quality in Healthcare (SECA) [16]. The scientific committee reviewed the individual index cards, validated the indicators, decided the formula for scoring them in real-world practice, and set the acceptable level of attainment for each of them. For example, for an indicator with a formula such as “Number of patients with HNC who initiate treatment × 100/Total number of patients diagnosed with HNC for whom treatment is indicated”, an acceptable level of ≥ 80% indicates that care is considered to be of good quality only if the resulting value is at least 80%.

### Statistical analysis

Delphi consensus was defined as at least two-thirds of Delphi respondents selecting a score sub-category that encompassed the median score of the group. Following RAND/UCLA guidelines, these score sub-categories were: 1–3 (inappropriate), 4–6 (undetermined), or 7–9 (appropriate) [17]. There was discordance among the Delphi panel when more than one-third of the experts scored within one sub-category, and more than one-third scored another. After the Delphi method was concluded, the scientific committee prioritised the indicators on which consensus was achieved by scoring them with a Likert-type scale from 1 (extremely inappropriate) to 5 (extremely appropriate). The mean and 95% confidence intervals (95% CI) were calculated for each of these indicators, ranking them and selecting those with the highest scores [18].

## Results

### Literature review and selection of indicators

**Fig. 2:**
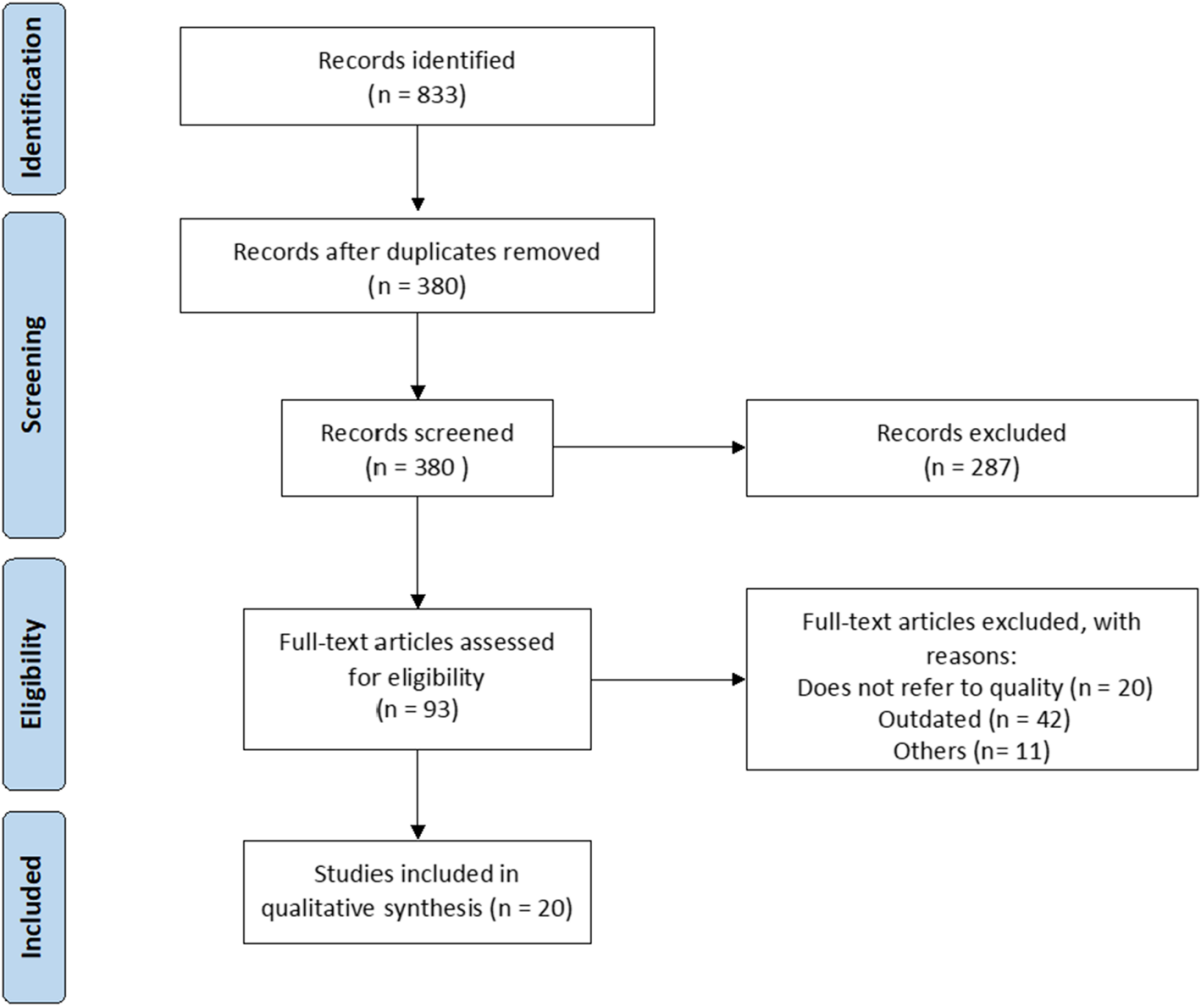
PRISMA flow diagram

The literature review yielded 833 documents, from which 20 were selected after removing duplicates and assessing relevance (Fig. 2). Initially, 44 indicators identified from the literature were presented to the scientific committee, which developed additional ones, resulting in a combined total of 50 indicators, covering diagnosis (13), treatment (28), follow-up (5), and health outcomes (4).

### Delphi study and development of indicators

The 50 indicators proposed by the scientific committee were then evaluated with a two-round Delphi method with participation from 52 experts in HNC in Spain. Participants were specialised in medical oncology, radiotherapy oncology, maxillofacial surgery, pathology, or otorhinolaryngology. The experts practiced in 11 of the 17 autonomous regions of Spain.

**Fig. 3:**
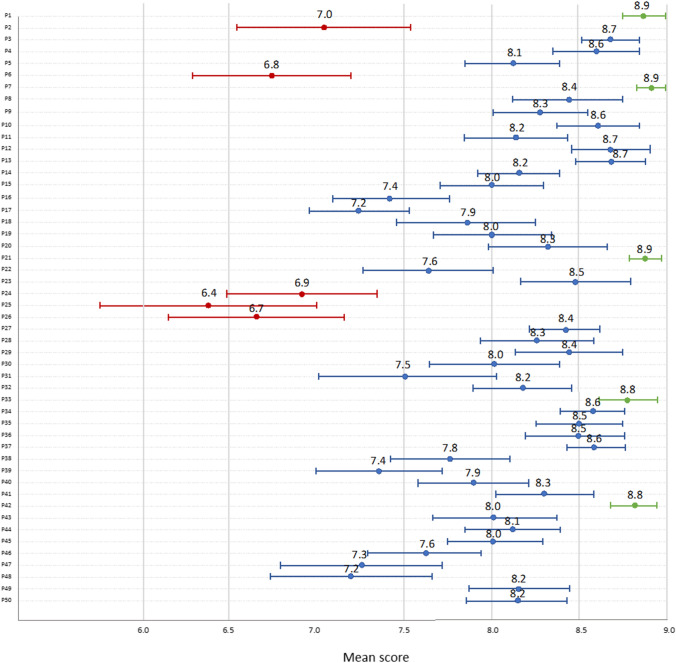
Delphi method scores of proposed criteria. Mean scores of proposed (P) indicators. Red, indicators with the lowest scores; blue, indicators with average scores; green, indicators with the highest scores. *P1* imaging study prior to initiating treatment, *P2* whole-body PET/CT up to 6 weeks prior to initiating treatment in patients with stage III–IV disease, *P3* whole-body PET/CT in tumours of unknown primary up to 6 weeks prior to initiating treatment, *P4* access to an anatomic pathology service for immunohistochemistry, *P5* access to a biomarker evaluation service, *P6* Narrow Band Imaging endoscopy, *P7* histological evaluation prior to initiating treatment, *P8* obtain anatomopathological report that includes TNM staging and biomarker information, *P9* routine evaluation of Epstein–Barr virus (EBV) and human papillomavirus (HPV) in lymphadenopathy of patients with metastatic head and neck squamous cell carcinoma of unknown primary, *P10* HPV evaluation and complete TNM staging prior to initiating treatment in patients with oropharyngeal cancer, *P11* EBV evaluation and complete TNM staging prior to initiating treatment in patients with cavum carcinoma, *P12* existence of a multidisciplinary Tumour Board, *P13* Tumour Board evaluation of histological diagnosis and TNM staging to determine the complete treatment plan using one document available to the team, *P14* assessment of patients’ nutritional status prior to initiating the first treatment, *P15* adequate oral cavity and dental assessment by an expert prior to initiating radiotherapy, *P16* availability of speech therapists, *P17* evaluation of all patients using validated comorbidity scales (e.g. Charlson Comorbidity Index) and/or performance status prior to initiating treatment, *P18* encourage smoker patients with head and neck cancer to start a cessation programme with a specialised clinician, *P19* encourage alcoholic patients with head and neck cancer to start a cessation programme with a specialised clinician, *P20* Tumour Board establishment of an optimal timeframe between selecting a therapy with curative intent and treatment initiation, *P21* complete tumour resection in patients who undergo surgery with curative intent, *P22* access to transoral surgical techniques (manual or robotic), *P23* maintain adequate surgical margins (≥ 3 mm and ≤ 5 mm) in patients with head and neck squamous cell carcinoma of the oral cavity larynx or pharynx who undergo open surgical resection with curative intent, *P24* re-hospitalisation within 30 days of surgery for surgery-related issues, *P25* repeat surgery within 7 days of the first surgery, *P26* long hospitalisation (≥ 30 days) after surgery, *P27* initiate adjuvant radiotherapy within 6 weeks of surgery, *P28* use of intensity-modulated radiotherapy in radical radiotherapy, *P29* chemoradiotherapy with cisplatin (tri-weekly or weekly) in patients with stage III or IV head and neck squamous cell carcinoma of the oral cavity larynx or pharynx with extracapsular spread and/or involved margins (< 1 mm), *P30* monotherapy (surgery or radiotherapy) for patients with early-stage head and neck squamous cell carcinoma, *P31* total laryngectomy in patients with head and neck squamous cell carcinoma of the larynx in stage T4a or with non-metastatic invasion of thyroid cartilage, *P32* radiotherapy concomitant with cisplatin or cetuximab for patients with locally advanced head and neck squamous cell carcinoma who are not eligible for surgery, *P33* evaluation of response to chemoradiotherapy using imaging (computed tomography or fluorodeoxyglucose–positron emission tomography) and physical examination (inspection of the oral cavity or nasofibroscopy) of patients with locally advanced head and neck cancer, *P34* evaluation of response to chemoradiotherapy in patients with locally advanced disease 8–12 weeks after treatment completion, *P35* access to immunotherapy by eligible patients with recurrent and/or metastatic disease, *P36* Tumour Board re-assessment of patients with local or systemic recurrence, *P37* multidisciplinary assessment of patients with recurrent oligometastasis to provide local therapy with radical intent (salvage therapy), *P38* second- or third-line therapy for eligible patients with advanced head and neck cancer who have not responded to previous lines of therapy or have recurrent disease, *P39* assessment of quality of life (using validated questionnaires) before and after treatment in patients with recurrent/metastatic disease who receive second- or third-line therapy, *P40* determination of PD-L1 expression using Combined Positive Score in patients with advanced disease, *P41* propose participation in clinical trials that fit the patient’s clinical characteristics and therapeutic needs, *P42* adequate follow-up after treatment completion, *P43* evaluate thyroid function every 6–12 months after neck irradiation, *P44* assessment of oral cavity and teeth in patients who have received radiotherapy in the oral cavity, *P45* patient follow-up by a multidisciplinary team to assess physical sequelae, *P46* patient follow-up by a multidisciplinary team to assess psychosocial sequelae, *P47* evaluate survival 30 and 90 days after surgery, *P48* evaluate survival 30 and 90 days after non-surgical therapy with radical intent, *P49* overall survival 1, 3 and 5 years from diagnosis, *P50* overall survival by stage 1, 3 and 5 years from diagnosis

With the first survey, 47 of the 50 proposed indicators achieved consensus. After the two rounds, consensus was achieved on the appropriateness of all 50 indicators (Fig. 3). The proposed (P) indicators that received the lowest scores (≤ 7) concerned the use of whole-body PET/CT (P2), use of Narrow Band Imaging endoscopy (P6), re-hospitalisation within 30 days of surgery for surgery-related issues (P24), repeat surgery within 7 days of the first surgery (P25), and long hospitalisation after surgery (P26). The indicators that received the highest scores (≥ 8.8) regarded the use of imaging before initiating treatment (P1), histological evaluation before initiating treatment (P7), complete resection of the tumour (P21), use of imaging to evaluate response to chemoradiotherapy (P33), and conducting an adequate follow-up after treatment completion (P42).

The scientific committee scored the 50 indicators to narrow down the list to those deemed most appropriate for use in clinical practice, resulting in a final selection of 29 indicators related to structure (3), process (22), or outcome (4) (Table 1). Easy-to-use index cards were developed for the 29 indicators, including their definition, formula, rationale, and acceptable level of attainment (Supplementary Material).

**Table 1:** List of recommended indicators for evaluating quality of care in head and neck cancer

No.	Topic	Dimension	Type	Measure
I1	Diagnosis	Diagnosis and staging	Process	Adequate imaging study prior to initiating treatment
I2	Diagnosis	Diagnosis and staging	Process	Whole-body PET/CT in tumours of unknown primary to determine the therapeutic strategy
I3	Diagnosis	Diagnosis and staging	Structure	Access to an anatomic pathology service for immunohistochemistry
I4	Diagnosis	Diagnosis and staging	Structure	Access to a biomarker evaluation service
I5	Diagnosis	Diagnosis and staging	Process	Histopathological study prior to initiating treatment
I6	Diagnosis	Diagnosis and staging	Process	Complete staging with TNM system prior to initiating treatment
I7	Diagnosis	Diagnosis and staging	Process	Routine evaluation of Epstein–Barr virus and human papillomavirus
I8	Diagnosis	Multidisciplinary care	Structure	Existence of a multidisciplinary Tumour Board
I9	Diagnosis	Considerations before treatment	Process	Multidisciplinary assessment prior to initiating treatment
I10	Treatment	Considerations before treatment	Process	Assessment of patients’ nutritional status prior to initiating treatment
I11	Treatment	Considerations before treatment	Process	Adequate oral cavity and dental assessment by an expert prior to initiating radiotherapy
I12	Treatment	Responsiveness	Process	Initiate treatment with curative intent within 14 days of the therapeutic decision
I13	Treatment	Surgery	Process	Complete tumour resection in patients undergoing surgery with curative intent
I14	Treatment	Reaction capacity	Process	Initiate treatment with adjuvant radiotherapy within 6 weeks of surgery
I15	Treatment	Radiotherapy	Process	Use of intensity-modulated radiotherapy in radical radiotherapy
I16	Treatment	Early stages	Process	Adequate use of monotherapy in the early stages of the disease
I17	Treatment	Stages III and IV	Process	Adjuvant chemoradiotherapy with cisplatin in patients with stage III or IV HNSCC
I18	Treatment	Locally advanced	Process	Adequate indication for chemoradiotherapy in patients with locally advanced disease
I19	Treatment	Follow-up of locally advanced	Outcome	Assessment of response to chemoradiotherapy after completing radical treatment in patients with locally advanced (stage III, IVA and IVB) disease
I20	Treatment	Follow-up of locally advanced	Outcome	Assessment of response to chemoradiotherapy in locally advanced patients (stage III, IVA and IVB) 8–12 weeks after radical treatment completion
I21	Treatment	Considerations before treatment	Process	Determination of PD-L1 expression
I22	Treatment	Recurrent and/or metastatic	Process	Access to immunotherapy by eligible patients with recurrent and/or metastatic disease
I23	Treatment	Recurrent and/or metastatic	Process	Tumour board assessment of patients with local or systemic recurrence
I24	Treatment	Recurrent and/or metastatic	Process	Assessment of second- or third-line therapy in patients with recurrent and/or meta-static head and neck cancer
I25	Treatment	Inclusion in clinical trials	Process	Proposal of participation in clinical trials
I26	Follow-up	–	Process	Adequate follow-up after treatment completion
I27	Follow-up	–	Process	Evaluation of thyroid function after neck irradiation
I28	Health outcomes	–	Outcome	Mortality after surgery
I29	Health outcomes	–	Outcome	Mortality after non-surgical treatment with radical intent

*HNSCC* head and neck squamous cell carcinoma, *PET/CT* positron emission tomography/computed tomography, *TNM* tumour, node, metastasis

## Discussion

We have developed 29 evidence-based and expert-supported indicators for evaluating the quality of care in HNC. These indicators assess quality at various steps of the patient cancer journey, from diagnosis to follow-up. Some of the indicators concern specific stages or tumour types, enabling a more accurate evaluation of care that takes into account particularities that may not be applicable to all HNC.

The National Comprehensive Cancer Network recently reviewed and endorsed a set of measures to evaluate the quality of cancer care, with seven cross-cancer measures and a few additional ones specific to breast, colorectal, lung, or prostate cancers, but not HNC [19]. QOPI^®^ by ASCO also lacks measures that are specific to HNC [20]. Although the quality of care for patients with HNC has increased over time [6], there is still room for improvement [6–8]. To adequately assess and improve the quality of care, indicators are needed to adequately cover the steps involved in patient management. Some approaches have been developed for improving the quality of surgery in HNC [21] but there is a general scarcity of data on the development and/or implementation of initiatives that aim to improve quality in the spectrum of HNC care [22]. Success in certain aspects has been described, such as timely initiation of radiotherapy after surgery [23] or improved quality of radiotherapy [24].

Two large studies in the USA found broad variability in adherence to five quality-of-care measures in patients with HNSCC [6, 7] regardless of patient volume and safety-net burden [7]. Adherence ranged from 44.5% in the case of initiation of adjuvant therapy within 6 weeks to 80.0% in the case of negative margins [6]. Patients who received high-quality care—defined as over 75% adherence to the measures—had a 19% reduced risk of mortality [6]. All five measures were independently associated with a reduced risk of mortality [6]. Four of these measures are included in the list of 29 indicators we developed here for use in clinical practice: negative surgical margins (I13, complete tumour resection in patients undergoing surgery with curative intent), appropriate adjuvant radiation and appropriate adjuvant chemoradiation (I17, adjuvant chemoradiotherapy with cisplatin in patients with stage III or IV HNSCC; I18, adequate indication of chemoradiotherapy in patients with locally advanced disease), and postoperative adjuvant therapy within 6 weeks (I14, delay of radiotherapy after surgery). The fifth measure considered by Cramer et al. [6] was neck dissection yield ≥ 18 lymph nodes; we did not include this as an indicator in our study, given the variability of lymph nodes that may be involved depending on the patient and type of tumour.

Use of quality measures and indicators can improve healthcare in several ways, by enabling objective monitoring of the quality of care and making auditing possible, to identify differences in patterns of care and benchmark departments/hospitals. Audits and feedback impact patient care and improve healthcare practice [25, 26]. Barriers to quality of care must be considered, including workload, lack of clarity on accountability, and lack of coordination of care [27], and factors that impact the success of feedback should also be taken into account [28]. A risk adjustment is needed when implementing measures related to outcomes and making comparisons to ensure that the differences found are due to differences in quality of care and not to other causes, such as increased workload [29].

This study has several strengths. First, a systematic approach was followed to develop the indicators recommended here, reviewing the appropriate literature, using a Delphi method, and prioritising indicators to select those most fitting for use in clinical practice. Second, the indicators were developed by a group of oncologists, and the panel of experts who participated in the Delphi method was multidisciplinary, including specialties other than oncology, such as surgery, pathology, or otorhinolaryngology. The main limitation of this study is the potentially challenging wide-scale use of the recommended indicators, given the underlying multidisciplinary coordination required among healthcare professionals as well as the disparate organisation of healthcare systems within and between countries. Although the indicators were developed by clinicians practicing in Spain, the literature review was not restricted to one country; as such, the indicators should have ample applicability, with potential tailoring to the specific needs of each centre or health system. Another limitation is the omission of criteria and indicators concerning dosimetry or toxicity follow-up. While these topics are of interest in the overall scope of quality of care, the criteria developed here have a more general character and specific details on doses or approaches to classify toxicity were not considered. Laryngectomy rates were also not taken into consideration because of the high variability between centres in the therapeutic approach used to preserve the larynx, given the ongoing debate on this matter.

The HNC treatment landscape is constantly evolving, and we suggest updating the indicators presented here every 2–3 years to reflect both advances in the field and patient needs. In particular, novel combinations of immunotherapy with other agents are being evaluated in clinical trials as well as the use of immunotherapy in locally advanced disease and in the adjuvant or neoadjuvant setting [5, 30, 31]; findings from these studies may change the treatment algorithm in the near future. Studies that evaluate the impact of initiatives that implement quality-of-care indicators are needed. We aim for the indicators developed here to drive improvement of care for patients with HNC. If there are challenges in meeting the quality standards according to the indicators we have developed, it may be preferable to treat patients at tertiary referral centres. We also hope that these indicators encourage healthcare professionals to evaluate the quality of care at their respective centres with the ultimate goal of delivering high-quality care.

### Supplementary Information

Below is the link to the electronic supplementary material.

Supplementary file1 (DOCX 1425 KB)

## Data Availability

Not applicable.
