# Supplementary material for: Indicators to evaluate quality of care in head and neck cancer in Spain
Source: Clin Transl Oncol. 2023 Oct 17;26(5):1089–97. doi: 10.1007/s12094-023-03298-z (PMC11026290; doi:10.1007/s12094-023-03298-z)
Supplement: Supplementary file 1 — Supplementary file1 (DOCX 1425 KB) [file 12094_2023_3298_MOESM1_ESM.docx]

1. **DIAGNOSIS**

**DIMENSION: DIAGNOSIS AND STAGING OF THE DISEASE**

| **Criterion 1: Adequate use of imaging in head and neck cancer** | **PRO-01** |
| --- | --- |
| **INDICATOR: Adequate imaging prior to initiation of treatment** | |
| **DEFINITION:** Evaluation of patients with head and neck cancer by diagnostic techniques including CT with contrast and/or MRI in the primary tumour area before initiating treatment. In addition, chest radiography and chest CT should be performed depending on the stage of the tumour. | |
| **FORMULA:** Number of patients with head and neck cancer who have had appropriate imaging prior to initiating treatment x 100 / Total number of patients diagnosed with head and neck cancer for whom treatment is indicated. | |
| **INDICATOR of:** Process | |
| **RATIONALE / EXCLUSIONS / CLARIFICATIONS:**  RATIONALE:  A precise diagnosis of the spread of the disease using imaging is key in planning adequate treatment.  EXCLUSIONS:  - Patients who die before starting the first treatment.  - Patients who refuse to undergo imaging.  CLARIFICATIONS:   - - In primary location and lymphatic drainage area, CT with contrast and/or MRI should be performed to evaluate the primary tumour and regional lymph nodes, as well as cartilage invasion in laryngeal or hypopharyngeal cancer.   - In stages I and II, a chest X-ray should be performed to rule out lesions.   - In stages III and IV a thoracic CT scan should be performed to determine tumour extension and guide therapeutic decisions.   CT, computed tomography; MRI, magnetic resonance imaging | |
| **SOURCE OF INFORMATION:** Clinical records. | |
| **CALCULATION PERIOD:** The last 12 months. | |
| **ACCEPTABLE LEVEL: ≥95**% | |
| **REFERENCES:**   - NHS Scottish Cancer Taskforce National Cancer Quality Steering Group Final Head and Neck Cancer QPI Document v3.0 and v 4.0– (13th July 2018). - Leroy R, Gendt C, Stordeur S, Schillemans V, Verleye L, Silversmit G, Van Eycken E, Savoye I, Grégoire V, Nuyts S and Vermorken J. Head and Neck Cancer in Belgium: Quality of Diagnostic Management and Variability Across Belgian Hospitals Between 2009 and 2014. Front. Oncol. 2019:1006. doi: 10.3389/fonc.2019.01006. - Machiels JP, René Leemans C, Golusinkiw W, Grau C, Liatra L and Gregorie V on behalf of the EHNS Executive Board, ESMO Guidelines Committee and ESTRO Executive Board. Squamous cell carcinoma of the oral cavity, larynx, oropharynx and hypopharynx: ENS-ESMO-ESTRO Clinical Practice Guidelines for diagnosis, treatment and follow-up. Annals of Oncology. 2020 Nov;31(11):1462-1475. doi: 10.1016/j.annonc.2020.07.011. - NCCN Clinical Practice Guidelines in Oncology (NCCN Guidelines®). Head and Neck Cancers V3.2021. | |

**DIMENSION: DIAGNOSIS AND STAGING OF THE DISEASE**

| **Criterion 2: Appropriate use of imaging tests in head and neck cancer** | **PRO-02** |
| --- | --- |
| **INDICATOR: Whole-body PET/CT in tumours of unknown primary to determine the therapeutic strategy** | |
| **DEFINITION:** Whole-body PET/CT should be performed on tumours of unknown primary prior to initiating treatment. | |
| **FORMULA:** Number of patients with head and neck cancer with squamous histology of unknown primary who undergo whole-body PET/CT prior to initiating treatment x 100 / Total number of patients diagnosed with head and neck cancer with tumour of unknown primary. | |
| **INDICATOR of:** Process | |
| **RATIONALE / EXCLUSIONS / CLARIFICATIONS:**  RATIONALE:  Whenever CT, MRI and nasofibroscopy do not reveal the origin of the primary tumour, a PET/CT should be conducted to determine the tumour origin and the therapeutic strategy.  EXCLUSIONS:   - Patients who die before starting the first treatment. - Patients who refuse to undergo imaging.   CLARIFICATIONS:  Period for imaging is 6 weeks prior to initiating treatment.  CT, computed tomography; MRI, magnetic resonance imaging; PET, positron emission tomography | |
| **SOURCE OF INFORMATION:** Clinical records. | |
| **CALCULATION PERIOD**: The last 12 months. | |
| **ACCEPTABLE LEVEL: ≥80%.** | |
| **REFERENCES:**   - Leroy R, Gendt C, Stordeur S, Schillemans V, Verleye L, Silversmit G, Van Eycken E, Savoye I, Grégoire V, Nuyts S and Vermorken J. Head and Neck Cancer in Belgium: Quality of Diagnostic Management and Variability Across Belgian Hospitals Between 2009 and 2014. Front. Oncol. 2019:1006. doi: 10.3389/fonc.2019.01006. - NCCN Clinical Practice Guidelines in Oncology (NCCN Guidelines®). Head and Neck Cancers V3.2021. | |

**DIMENSION: DIAGNOSIS AND STAGING OF THE DISEASE**

| **Criterion 3: Immunohistochemical evaluation of head and neck cancer.** | **STR-01** |
| --- | --- |
| **INDICATOR: Access to an Anatomical Pathology Service that performs immunohistochemistry.** | |
| **DEFINITION:** Adequate access to an anatomical pathology service to perform immunohistochemistry testing and determine the presence of HPV infection, EBV and PD-L1 expression in order to properly evaluate and treat patients with head and neck cancer. | |
| **FORMULA:** Access to immunohistochemistry on tumour tissue of patients with head and neck squamous cell carcinoma in an in-house or contracted Anatomical Pathology service. | |
| **INDICATOR of:** Structure | |
| **RATIONALE / EXCLUSIONS / CLARIFICATIONS**:  RATIONALE:  Immunohistochemistry in patients with head and neck cancer enables evaluating the presence of HPV and/or EBV infection. PD-L1 expression can also be evaluated in tumour cells and in the tumour microenvironment.  EXCLUSIONS:   - Insufficient histological material to perform immunohistochemistry.   CLARIFICATIONS:  EBV should be evaluated in patients with nasopharyngeal carcinoma using in situ hybridisation for EBV-encoded RNA; HPV should be evaluated in patients with oropharyngeal carcinoma staining for p16; PD-L1 should be evaluated if immunotherapy is indicated.  EBV, Epstein­–Barr virus; HPV, human papillomavirus; PD-L1, programmed death ligand 1. | |
| **SOURCE OF INFORMATION:** Hospital / Department. | |
| **CALCULATION PERIOD**: The last 12 months. | |
| **ACCEPTABLE LEVEL:** Access. | |
| **REFERENCES:**   - NCCN Clinical Practice Guidelines in Oncology (NCCN Guidelines®). Head and Neck Cancers V3.2021. - Machiels JP, René Leemans C, Golusinkiw W, Grau C, Liatra L and Gregorie V on behalf of the EHNS Executive Board, ESMO Guidelines Committee and ESTRO Executive Board. Squamous cell carcinoma of the oral cavity, larynx, oropharynx and hypopharynx: ENS-ESMO-ESTRO Clinical Practice Guidelines for diagnosis, treatment and follow-up. Annals of Oncology. 2020 Nov;31(11):1462-1475. doi: 10.1016/j.annonc.2020.07.011. | |

**DIMENSION: DIAGNOSIS AND STAGING OF THE DISEASE**

| **Criterion 4: Access to studies in molecular biology** | **STR-02** |
| --- | --- |
| **NAME OF INDICATOR: Access to a biomarker evaluation service.** | |
| **DEFINITION:** Adequate access to molecular biology studies and evaluation of biomarkers of interest, in the same centre or in a different one. | |
| **FORMULA:** Access to molecular biology tests and biomarkers evaluation of the tumour tissue of patients with head and neck squamous cell carcinoma, either in the same centre or a different one. | |
| **INDICATOR of:** Structure | |
| **RATIONALE / EXCLUSIONS / CLARIFICATIONS:**  RATIONALE:  Biomarkers enable classifying head and neck squamous cell carcinoma and, in some cases, determining the treatment strategy.  CLARIFICATIONS:  This indicator will be of special interest to evaluate patients with salivary gland cancer. | |
| **SOURCE/S OF INFORMATION.** Hospital / Department. | |
| **CALCULATION PERIOD**: The last 12 months. | |
| **ACCEPTABLE LEVEL:** Access. | |
| **REFERENCES:**   - NCCN Clinical Practice Guidelines in Oncology (NCCN Guidelines®). Head and Neck Cancers V3.2021. - Machiels JP, René Leemans C, Golusinkiw W, Grau C, Liatra L and Gregorie V on behalf of the EHNS Executive Board, ESMO Guidelines Committee and ESTRO Executive Board. Squamous cell carcinoma of the oral cavity, larynx, oropharynx and hypopharynx: ENS-ESMO-ESTRO Clinical Practice Guidelines for diagnosis, treatment and follow-up. Annals of Oncology. 2020 Nov;31(11):1462-1475. doi: 10.1016/j.annonc.2020.07.011. | |

**DIMENSION: DIAGNOSIS AND STAGING OF THE DISEASE**

| **Criterion 5: Histopathologic evaluation of head and neck cancer.** | **PRO-03** |
| --- | --- |
| **INDICATOR: Histopathological study prior to initiating treatment in patients with head and neck cancer.** | |
| **DEFINITION:** Histological study should be conducted on the biopsy of the primary tumour prior to the first treatment and the affected lymph nodes, if present, to establish the definitive diagnosis before initiating treatment. | |
| **FORMULA:** Number of patients with head and neck cancer undergoing histological study before initiating treatment x 100 / Total number of patients diagnosed with head and neck cancer for whom treatment is indicated. | |
| **INDICATOR of:** Process | |
| **RATIONALE / EXCLUSIONS / CLARIFICATIONS:**  RATIONALE: The histological study of the biopsy confirms diagnostic malignancy before starting treatment. An appropriate sample is required to conduct relevant immunohistochemistry.  EXCLUSIONS:   - Patients who die before starting the first treatment. - Patients who refuse to undergo biopsy. - Patients who must undergo urgent surgery before receiving immunohistochemistry results.   CLARIFICATIONS:  The histological study of head and neck cancer specimens must be performed by an anatomical pathologist who is familiar with the World Health Organization classification recommendations for head and neck cancer. | |
| **SOURCE OF INFORMATION:** Clinical records. | |
| **CALCULATION PERIOD**: The last 12 months. | |
| **ACCEPTABLE LEVEL: ≥95%.** | |
| **REFERENCES:**   - Machiels JP, René Leemans C, Golusinkiw W, Grau C, Liatra L and Gregorie V on behalf of the EHNS Executive Board, ESMO Guidelines Committee and ESTRO Executive Board. Squamous cell carcinoma of the oral cavity, larynx, oropharynx and hypopharynx: ENS-ESMO-ESTRO Clinical Practice Guidelines for diagnosis, treatment and follow-up. Annals of Oncology. 2020 Nov;31(11):1462-1475. doi: 10.1016/j.annonc.2020.07.011. - El-Naggar AK, Chan JKC, Grandis JR, Takata T, Slootweg PJ. WHO Classification of Head and Neck Tumours. 2017. WHO Classification of Tumours, 4^th^ Edition, Volume 9. - NHS Scottish Cancer Taskforce National Cancer Quality Steering Group Final Head and Neck Cancer QPI Document v3.0 and v 4.0– (13^th^ July 2018). - Quality Oncology Reporting Initiative. QOPI® 2020 REPORTING TRACKS. | |

**DIMENSION: DIAGNOSIS AND STAGING OF THE DISEASE**

| **Criterion 6: Appropriate use of diagnostic tests in head and neck cancer** | **PRO-04** |
| --- | --- |
| **INDICATOR: Complete staging with TNM system prior to initiating treatment** | |
| **DEFINITION:** Complete staging with the TNM system before initiating treatment, including type of lesion; it should include size, location, and extension to other organs (neighbouring or distant). | |
| **FORMULA:** Number of patients with head and neck cancer staged using TNM before initiating treatment x 100 / Total number of patients diagnosed with head and neck cancer for whom treatment is indicated. | |
| **INDICATOR of:** Process | |
| **RATIONALE / EXCLUSIONS / CLARIFICATIONS:**  RATIONALE:  The complete diagnostic and classification report patients with head and neck cancer should include TNM classification, histology report, and immunohistochemistry and biomarker testing, if required.  EXCLUSIONS:   - Patients who die before initiating the first treatment. - Patients who refuse to undergo diagnostic tests. - Patients not undergoing active cancer treatment.   CLARIFICATIONS:  Perform complete staging with the TNM system (validated by the Tumour Board, if possible) before treatment. In addition, the following information is needed:   - - Location, larger diameter, lymphovascular infiltration   - Immunohistochemistry   - Invasion of nearby structures   - In oropharynx: HPV in oropharynx   - Adenopathy of unknown origin: HPV by in *situ* hybridisation and EBV.   - PD- L1 in advanced stages   TNM:   - T is the primary tumour - N are the affected lymph nodes - M are distant metastases   EBV, Epstein-Barr Virus; HPV, human papillomavirus; TNM, tumour, node, metastasis; PD-L1, programmed cell death ligand 1. | |
| **SOURCE OF INFORMATION:** Clinical records. | |
| **CALCULATION PERIOD**: The last 12 months. | |
| **ACCEPTABLE LEVEL:** 100%. | |
| **REFERENCES:**   - Quality Oncology Reporting Initiative. QOPI® 2020 REPORTING TRACKS. - NCCN Clinical Practice Guidelines in Oncology (NCCN Guidelines®). Head and Neck Cancers V3.2021. - Machiels JP, René Leemans C, Golusinkiw W, Grau C, Liatra L and Gregorie V on behalf of the EHNS Executive Board, ESMO Guidelines Committee and ESTRO Executive Board. Squamous cell carcinoma of the oral cavity, larynx, oropharynx and hypopharynx: ENS-ESMO-ESTRO Clinical Practice Guidelines for diagnosis, treatment and follow-up. Annals of Oncology. 2020 Nov;31(11):1462-1475. doi: 10.1016/j.annonc.2020.07.011. - O’Sullivan B. Head and neck tumours. In: Brierley J,Gospodarowicz MK, Wittekind C, eds. UICC TNM Classification of Malignant Tumours. 8 ed. Chichester: Wiley; 2017:17-54. - Shah JP and Montero PH. New AJCC/UICC staging system for head and neck, and thyroid cancer. 2018; 29(4) 397-404. doi: 10.1016/j.rmclc.2018.07.002. - Amin MB, Greene FL, Edge SB et al. (2017). The eighth edition AJCC cancer staging manual: continuing to build a bridge from a population‐based to a more “personalized” approach to cancer staging. CA: a cancer journal for clinicians, 67(2), 93-99. doi: 10.3322/caac.21388. | |

**DIMENSION: DIAGNOSIS AND STAGING OF THE DISEASE**

| **Criterion 7: Appropriate use of diagnostic tests in head and neck cancer** | **PRO-05** |
| --- | --- |
| **INDICATOR: Routine evaluation of Epstein–Barr virus and human papillomavirus** | |
| **DEFINITION:** HPV and EBV testing in lymphadenopathy in the neck of patients with metastatic head and neck squamous cell carcinoma of unknown primary. | |
| **FORMULA:** Number of patients with metastatic head and neck squamous cell carcinoma of unknown primary who are tested for HPV and EBV in lymphatic tissue x 100 / Total number of patients with metastatic head and neck squamous cell carcinoma of unknown primary. | |
| **INDICATOR of:** Process | |
| **RATIONALE / EXCLUSIONS / CLARIFICATIONS:**  RATIONALE:  The use of HPV and EBV testing can help determine the origin of an unknown primary tumour in patients with squamous cell cancer.  EXCLUSIONS:   - Patients who die before starting the first treatment. - Patients who refuse to undergo diagnostic tests. - Patients not undergoing active oncologic treatment.   CLARIFICATIONS:  p16 determined by immunohistochemistry is a reliable surrogate marker of positive oropharyngeal HPV. p16 positivity is defined as ≥70% nuclear and cytoplasmic expression with at least moderate to strong intensity. If head and neck squamous cell carcinoma of unknown primary is p16 positive, diagnosis should be confirmed by evaluation of DNA or RNA or by in situ hybridisation (ISH).  To rule out nasopharyngeal cancer, EBV infection should be determined using ISH for EBV-encoded RNA .  DNA, deoxyribonucleic acid; EBV, Epstein-Barr virus; HPV, human papillomavirus**;** ISH, in situ hybridisation; RNA, ribonucleic acid. | |
| **SOURCE OF INFORMATION:** Clinical records. | |
| **CALCULATION PERIOD**: The last 12 months. | |
| **ACCEPTABLE LEVEL: ≥90%.** | |
| **REFERENCES:**   - NCCN Clinical Practice Guidelines in Oncology (NCCN Guidelines®). Head and Neck Cancers V3.2021. - Machiels JP, René Leemans C, Golusinkiw W, Grau C, Liatra L and Gregorie V on behalf of the EHNS Executive Board, ESMO Guidelines Committee and ESTRO Executive Board. Squamous cell carcinoma of the oral cavity, larynx, oropharynx and hypopharynx: ENS-ESMO-ESTRO Clinical Practice Guidelines for diagnosis, treatment and follow-up. Annals of Oncology. 2020 Nov;31(11):1462-1475. doi: 10.1016/j.annonc.2020.07.011. - Fakhry C, Lacchetti C, Rooper LM, et al. Human papillomavirus testing in head and neck carcinomas: ASCO Clinical Practice Guideline endorsement of the College of American Pathologists guideline. J Clin Oncol. 2018;36(31):3152-3161. doi: 10.1200/JCO.18.00684. - Rassy E, Nicolai P, Pavlidis N. Comprehensive management of HPV related squamous cell carcinoma of the head and neck of unknown primary. Head Neck. 2019;41(10):3700-3711. doi: 10.1002/hed.25858. - Cohen EEW, Bell RB, Bifulco CB, et al. The Society for Immunotherapy of Cancer consensus statement on immunotherapy for the treatment of squamous cell carcinoma of the head and neck (HNSCC). J Immunother Cancer. 2019;7(1):184. doi: 10.1186/s40425-019-0662-5. - The Expert Panel on HPV Testing in Head and Neck Squamous Cell Carcinoma. Routine HPV Testing in Head and Neck Squamous Cell Carcinoma: Guideline Recommendations. A Quality Initiative of the Program in Evidence-Based Care (PEBC), Cancer Care Ontario (CCO) January 2020. Evidence-Based Series 5-9: Section 1. | |

**DIMENSION: MULTIDISCIPLINARY CARE**

| **Criterion 8: Multidisciplinary care for patients with head and neck cancer** | **STR-03** |
| --- | --- |
| **INDICATOR: Existence of a Multidisciplinary Tumour Board** | |
| **DEFINITION:** Every hospital that treats head and neck cancer must have specific committees for the evaluating patients prior to therapeutic decisions; these committees should include all professionals involved in the diagnostic and therapeutic process. | |
| **FORMULA:** Availability of an in-person or virtual multidisciplinary tumour board at the centre that integrates professionals involved in diagnosis, treatment, and follow-up of patients with head and neck carcinoma. | |
| **INDICATOR of:** Structure | |
| **RATIONALE / EXCLUSIONS / CLARIFICATIONS:**  RATIONALE:  Head and neck cancer is a complex disease that requires therapeutic interventions from different specialists, who must coordinate properly. Therefore, before initiating treatment, the patient should be evaluated by a multidisciplinary team and the treatments to be performed should be agreed upon by the different specialists. Scientific evidence suggests that patient management by a multidisciplinary team leads to better health outcomes and patient satisfaction.  CLARIFICATIONS:  The Head and Neck Cancer Tumour Board should be a multidisciplinary team comprising:   - Medical Oncologists - Head and Neck Surgeons (ENT and/or maxillofacial) - Radiation Oncologists - Radiologists and/or nuclear physicians - Anatomical pathologists - Other possible members to consider could be: nutrition specialists, psychologists, plastic surgeons, dentists, palliative care specialists, and nurse case managers.   ENT, ear, nose, and throat doctor. | |
| **SOURCE OF INFORMATION:** Hospital / Department. | |
| **CALCULATION PERIOD**: The last 12 months. | |
| **ACCEPTABLE LEVEL:** To have a tumour board comprising the abovementioned specialties. | |
| **REFERENCES:**   - Van Overveld LFJ, Braspenning JCC, Hermens RPMG. Quality indicators of integrated care for patients with head and neck cancer. Clin. Otolaryngol. 2017, 42, 322–329. doi: 10.1111/coa.12724. - NCCN Clinical Practice Guidelines in Oncology (NCCN Guidelines®). Head and Neck Cancers V3.2021. - Takes RP, Halmos GB, Ridge JA, Bossi P, Merkx MAW, Rinaldo A, Sanabria A, Smeele LE, Mäkitie AA, Ferlito A. Value and Quality of Care in Head and Neck Oncology. Current Oncology Reports (2020) 22: 92. [doi: 10.1007/s11912-020-00952-5](https://doi.org/10.1007/s11912-020-00952-5). - NHS Scottish Cancer Taskforce National Cancer Quality Steering Group Final Head and Neck Cancer QPI Document v3.0 and v 4.0– (13th July 2018). - Cristiana Lo Nigro C, Denaro N, Merlotti A y Merlano M. Head and neck cancer: improving outcomes with a multidisciplinary approach. Cancer Management and Research 2017:9 363–371. doi: 10.2147/CMAR.S115761. - Ministerio de Sanidad y Política Social. Estrategia en Cáncer del Sistema Nacional de Salud. 2021. <https://www.mscbs.gob.es/organizacion/sns/planCalidadSNS/pdf/Estrategia_en_cancer_del_Sistema_Nacional_de_Salud_Actualizacion_2021.pdf> - Machiels JP, René Leemans C, Golusinkiw W, Grau C, Liatra L and Gregorie V on behalf of the EHNS Executive Board, ESMO Guidelines Committee and ESTRO Executive Board. Squamous cell carcinoma of the oral cavity, larynx, oropharynx and hypopharynx: ENS-ESMO-ESTRO Clinical Practice Guidelines for diagnosis, treatment and follow-up. Annals of Oncology. 2020 Nov;31(11):1462-1475. doi: 10.1016/j.annonc.2020.07.011. | |

**DIMENSION: ACTIONS TO BE TAKEN BEFORE STARTING TREATMENT**

| **Criterion 9: Multidisciplinary care for patients with head and neck cancer** | **PRO-06** |
| --- | --- |
| **INDICATOR: Multidisciplinary assessment prior to initiating treatment** | |
| **DEFINITION:** All patients with head and neck cancer should be evaluated by the Tumour Board prior to initiating treatment to ensure histological diagnosis, establish TNM, and develop a complete treatment plan to be made available to the team in one document (clinical record or report from the Tumour Board). | |
| **FORMULA:** Number of patients with head and neck cancer evaluated by the Tumour Board prior to initiating treatment x 100 / Total patients with head and neck cancer. | |
| **INDICATOR of:** Process | |
| **RATIONALE / EXCLUSIONS / CLARIFICATIONS:**  RATIONALE:  Management (diagnosis, treatment, and follow-up) of patients with head and neck cancer should be discussed by a multidisciplinary team with extensive experience in this therapeutic area.  CLARIFICATIONS:  The Head and Neck Cancer Tumour Board will be a multidisciplinary team (meeting in person or virtually) comprising at least the following specialists:   - Medical Oncologists - Head and Neck Surgeons (ENT and/or maxillofacial) - Radiation Oncologists - Radiologist and/or nuclear physicians - Anatomical pathologists - Other possible members to consider could be: nutrition specialists, psychologists, plastic surgeons, dentists, palliative care specialists, and nurse case managers.   ENT, ear, nose, and throat doctor. | |
| **SOURCE OF INFORMATION:** Clinical records. | |
| **CALCULATION PERIOD**: The last 12 months. | |
| **ACCEPTABLE LEVEL:** 100%. | |
| **REFERENCES:**   - Takes RP, Halmos GB, Ridge JA, Bossi P, Merkx MAW, Rinaldo A, Sanabria A, Smeele LE, Mäkitie AA, Ferlito A. Value and Quality of Care in Head and Neck Oncology. Current Oncology Reports (2020) 22: 92. [doi: 10.1007/s11912-020-00952-5](https://doi.org/10.1007/s11912-020-00952-5). - NHS Scottish Cancer Taskforce National Cancer Quality Steering Group Final Head and Neck Cancer QPI Document v3.0 and v 4.0– (13^th^ July 2018). - Cristiana Lo Nigro C, Denaro N, Merlotti A y Merlano M. Head and neck cancer: improving outcomes with a multidisciplinary approach. Cancer Management and Research 2017:9 363–371. doi: 10.2147/CMAR.S115761. - Machiels JP, René Leemans C, Golusinkiw W, Grau C, Liatra L and Gregorie V on behalf of the EHNS Executive Board, ESMO Guidelines Committee and ESTRO Executive Board. Squamous cell carcinoma of the oral cavity, larynx, oropharynx and hypopharynx: ENS-ESMO-ESTRO Clinical Practice Guidelines for diagnosis, treatment and follow-up. Annals of Oncology. 2020 Nov;31(11):1462-1475. doi: 10.1016/j.annonc.2020.07.011. - Van Overveld LFJ, Braspenning JCC, Hermens RPMG. Quality indicators of integrated care for patients with head and neck cancer. Clin. Otolaryngol. 2017, 42, 322–329. doi: 10.1111/coa.12724. - ENT UK (2016) Head and Neck Cancer: Multidisciplinary Management Guidelines. Available from: <https://www.cambridge.org/core/journals/journal-of-laryngology-and-otology/head-and-neck-guidelines>. - Ministerio de Sanidad y Política Social. Estrategia en Cáncer de Sistema Nacional de Salud. 2010. <https://www.mscbs.gob.es/organizacion/sns/planCalidadSNS/pdf/ActualizacionEstrategiaCancer.pdf>. | |

1. **TREATMENT**

**DIMENSION: ACTIONS TO BE TAKEN BEFORE STARTING TREATMENT**

| **Criterion 10: Assessment of the patient's condition** | **PRO-07** |
| --- | --- |
| **INDICATOR: Assessment of patients’ nutritional status prior to initiating treatment** | |
| **DEFINITION:** Assessment of nutritional status using a validated tool in patients with head and neck cancer before initiating treatment. | |
| **FORMULA:** Number of patients with head and neck cancer whose nutritional status is assessed using a validated tool before starting treatment x 100 / Total number of patients with head and neck cancer for whom treatment is indicated. | |
| **INDICATOR of:** Process | |
| **RATIONALE / EXCLUSIONS / CLARIFICATIONS:**  RATIONALE:  Improving patients’ nutritional status is recommended before starting treatment in all patients, especially in those with an over 10% weight loss in the six months prior to diagnosis (significant malnutrition).  CLARIFICATIONS:  Nutritional support will be given orally if possible; if oral support is not possible, enteral nutrition will be provided, preferably via percutaneous endoscopic gastrostomy and not with nasogastric intubation when nutritional support is needed for a long period of time. | |
| **SOURCE OF INFORMATION:** Clinical records. | |
| **CALCULATION PERIOD**: The last 12 months. | |
| **ACCEPTABLE LEVEL: ≥85%.** | |
| **REFERENCES:**   - NHS Scottish Cancer Taskforce National Cancer Quality Steering Group Final Head and Neck Cancer QPI Document v3.0 and v 4.0– (13^th^ July 2018). - Machiels JP, René Leemans C, Golusinkiw W, Grau C, Liatra L and Gregorie V on behalf of the EHNS Executive Board, ESMO Guidelines Committee and ESTRO Executive Board. Squamous cell carcinoma of the oral cavity, larynx, oropharynx and hypopharynx: ENS-ESMO-ESTRO Clinical Practice Guidelines for diagnosis, treatment and follow-up. Annals of Oncology. 2020 Nov;31(11):1462-1475. doi: 10.1016/j.annonc.2020.07.011. - NCCN Clinical Practice Guidelines in Oncology (NCCN Guidelines®). Head and Neck Cancers V3.2021. - Nadershah M, Carlson ER, Young LS, Burke PA and Daley BJ. American Association of Oral and Maxillofacial Surgeons. {Nutritional Considerations for Head and Neck Cancer Patients: A Review of the Literature. J Oral Maxillofac Surg 71:1853-1860, 2013. doi: 10.1016/j.joms.2013.04.028. | |

**DIMENSION: ACTIONS TO BE TAKEN BEFORE STARTING TREATMENT**

| **Criterion 11: Assessment of the patient's condition** | **PRO-08** |
| --- | --- |
| **INDICATOR: Adequate oral cavity and dental assessment by an expert prior to initiating radiotherapy** | |
| **DEFINITION:** Assessment of the oral and dental status of patients with head and neck cancer and recommendation of a preventive treatment plan and follow-up. | |
| **FORMULA:** Number of patients with head and neck cancer with oral cavity and dental assessment who initiate radiotherapy x 100 / Total number of patients with head and neck cancer for whom radiotherapy is indicated. | |
| **INDICATOR of:** Process | |
| **RATIONALE / EXCLUSIONS / CLARIFICATIONS:**  RATIONALE:  Localised radiotherapy in the head and neck causes xerostomia and alters the function of the salivary glands, increasing the risk of caries and its sequelae, including dentoalveolar infections and osteoradionecrosis.  EXCLUSIONS:   - Patients with total edentulous for dental assessment. - Patients who have received radiotherapy with palliative intent. - Patients whose radiotherapy treatment does not affect the oral cavity or salivary glands.   CLARIFICATIONS:  Before starting treatment, an expert (e.g., dentist, maxillofacial surgeon) must evaluate the oral cavity and teeth, extract the teeth if needed, and prescribe preventive measures. | |
| **SOURCE OF INFORMATION:** Clinical records. | |
| **CALCULATION PERIOD**: The last 12 months. | |
| **ACCEPTABLE LEVEL: ≥80%.** | |
| **REFERENCES:**   - NCCN Clinical Practice Guidelines in Oncology (NCCN Guidelines®). Head and Neck Cancers V3.2021. - Machiels JP, René Leemans C, Golusinkiw W, Grau C, Liatra L and Gregorie V on behalf of the EHNS Executive Board, ESMO Guidelines Committee and ESTRO Executive Board. Squamous cell carcinoma of the oral cavity, larynx, oropharynx and hypopharynx: ENS-ESMO-ESTRO Clinical Practice Guidelines for diagnosis, treatment and follow-up. Annals of Oncology. 2020 Nov;31(11):1462-1475. doi: 10.1016/j.annonc.2020.07.011. - Leroy R, De Gent C, Stordeur S, Silvermit G, Verleye L et al. Quality Indicators for the management of Head and Neck Squamous Cell Carcinoma. Belgium Health Care Knowledge 2019 Report. - Van Overveld LFJ, Braspenning JCC, Hermens RPMG. Quality indicators of integrated care for patients with head and neck cancer. Clin. Otolaryngol. 2017, 42, 322–329. doi: 10.1111/coa.12724. - Takes RP, Halmos GB, Ridge JA, Bossi P, Merkx MAW, Rinaldo A, Sanabria A, Smeele LE, Mäkitie AA, Ferlito A. Value and Quality of Care in Head and Neck Oncology. Current Oncology Reports (2020) 22: 92. doi: 10.1007/s11912-020-00952-5. | |

**DIMENSION: RESPONSIVENESS**

| **Criterion 12: Responsiveness after the therapeutic decision.** | **PRO-09** |
| --- | --- |
| **INDICATOR: Initiate treatment with curative intent within 14 days of the therapeutic decision.** | |
| **DEFINITION:** Time elapsed from diagnostic confirmation to therapeutic decision by the Tumour Board to treatment initiation measured in calendar days. | |
| **FORMULA:** Number of patients with head and neck cancer with diagnostic confirmation and therapeutic decision who have initiated treatment within ≤14 calendar days x 100 / Total number of patients with head and neck cancer with diagnostic confirmation and therapeutic decision seen in the department. | |
| **INDICATOR of:** Process | |
| **RATIONALE / EXCLUSIONS / CLARIFICATIONS:**  RATIONALE:  Head and neck cancer arise in a challenging location and can lead to serious functional alterations; therefore, delay in treatment initiation may result in poor tumour control and patient quality of life.  EXCLUSIONS*:*   - Patients who die before initiating first treatment. - Patients who will receive only palliative care. - Patients who decide to not initiate treatment. - Patients lost to follow-up because they changed to a different centre.   CLARIFICATIONS:  The interval between the therapeutic decision and initiation of the recommended treatment is 14 calendar days. Therapeutic decision is the selection of the most appropriate treatment for the patient (e.g., pharmacological, surgical, radiotherapeutic) after tumour staging.  This criterion indirectly reflects the department’s ability to manage the patient workload with the available resources. | |
| **SOURCE OF INFORMATION:** Clinical records. | |
| **CALCULATION PERIOD**: The last 12 months. | |
| **ACCEPTABLE LEVEL: ≥70%** | |
| **REFERENCES:**   - Takes RP, Halmos GB, Ridge JA, Bossi P, Merkx MAW, Rinaldo A, Sanabria A, Smeele LE, Mäkitie AA, Ferlito A. Value and Quality of Care in Head and Neck Oncology. Current Oncology Reports (2020) 22: 92. [doi: 10.1007/s11912-020-00952-5](https://doi.org/10.1007/s11912-020-00952-5)**.** - Van Overveld LFJ, Braspenning JCC, Hermens RPMG. Quality indicators of integrated care for patients with head and neck cancer. Clin. Otolaryngol. 2017, 42, 322-329. doi: 10.1111/coa.12724. - Schutte HW, Heutink F, Wellenstein DJ, van der Broek GB et al. Time to Diagnosis and Treatment in Head and Neck Cancer: A Systematic Review. Otolaryngology– Head and Neck Surgery 2020, Vol. 162(4) 446–457. doi: 10.1177/0194599820906387. - Van Overveld LFJ, Takes RP, Smeele LE, et al. (2018) The Dutch Head and Neck Audit: The First Steps. J Head Neck Surg 1(1):1-8. doi:10.36959/605/528. - Leroy R, De Gent C, Stordeur S, Silvermit G, Verleye L et al. Quality Indicators for the management of Head and Neck Squamous Cell Carcinoma. Belgium Health Care Knowledge 2019 Report. | |

**DIMENSION: SURGICAL TREATMENT**

| **Criterion 13: Adequacy of surgical treatment** | **PRO-10** |
| --- | --- |
| **INDICATOR: Complete tumour resection in patients undergoing surgery with curative intent.** | |
| **DEFINITION:** Adequacy of surgical treatment in patients with head and neck squamous cell carcinoma of the oral cavity, larynx or pharynx undergoing surgery with curative intent. | |
| **FORMULA:** Number of patients with head and neck cancer undergoing surgery with curative intent and complete tumour excision with tumour-free surgical margins* x 100 / Total number of patients with head and neck cancer undergoing surgery with curative intent**.** | |
| **INDICATOR of:** Process | |
| **RATIONALE / EXCLUSIONS / CLARIFICATIONS:**  RATIONALE:  Achieving complete tumour resection in patients with head and neck cancer undergoing surgery with curative intent is associated with better locoregional disease control and increased overall survival.  EXCLUSIONS:   - Endoscopic surgeries in which the surgical technique complicates assessment of tumour margins.   CLARIFICATIONS:  Patients with head and neck cancer who undergo surgery with curative intent should follow a surgical plan with the goal of achieving total tumour resection and adequate tumour-free surgical margins. Surgical margins may be extended, if needed.  *Adequate surgical margins: ≥3 mm and ≤5 mm. | |
| **SOURCE OF INFORMATION:** Clinical records. | |
| **CALCULATION PERIOD**: The last 12 months. | |
| **ACCEPTABLE LEVEL: ≥90%** | |
| **REFERENCES:**   - NHS Scottish Cancer Taskforce National Cancer Quality Steering Group Final Head and Neck Cancer QPI Document v3.0 and v 4.0– (13^th^ July 2018). - NCCN Clinical Practice Guidelines in Oncology (NCCN Guidelines®). Head and Neck Cancers V3.2021. - Binahmed A, Nason R.W, Abdoh A.A (2007) The clinical significance of the positive surgical margin in oral cancer. Oral Oncology. 2007; 43: 780–784. doi: 10.1016/j.oraloncology.2006.10.001. - Wong L.S, McManon J, Devine J, McLellan D, Thompson E, Farrow A, Moosa K, Avoub A. (2012) Influence of close resection margins on local recurrence and disease-specific survival in oral and oropharyngeal carcinoma. British Journal of oral and maxillofacial surgery, 2012; 50: 102-108. doi: 10.1016/j.bjoms.2011.05.008. | |

**DIMENSION: RESPONSIVENESS**

| **Criterion 14: Responsiveness following the therapeutic decision** | **PRO-11** |
| --- | --- |
| **INDICATOR: Initiate treatment with adjuvant radiotherapy within 6 weeks of surgery** | |
| **DEFINITION:** Time elapsed from surgery to initiating adjuvant radiotherapy**.** | |
| **FORMULA:** Number of patients with head and neck cancer who initiate adjuvant radiotherapy within 6 weeks of surgery x 100 / Total number of patients with head and neck cancer undergoing radiotherapy after surgery. | |
| **INDICATOR of:** Process | |
| **RATIONALE / EXCLUSIONS / CLARIFICATIONS:**  RATIONALE:  The total time of radiotherapy treatment and the time elapsed from surgery to initiating radiotherapy impact local control of the disease and can indicate agility in the process.  EXCLUSIONS:   - Patients in whom radiotherapy is not indicated or is contraindicated. - Patients with surgically-derived complications that prevent initiation of radiotherapy.   CLARIFICATIONS:  Following recommendations from EHNS-ESMO-ESTRO 2020 and ASTRO 2017 guidelines, the total time from surgery to treatment completion should be ≤12 weeks.  The time elapsed from surgery to the start of adjuvant radiotherapy treatment should be under 6 weeks.  ASTRO, American Society for Radiation Oncology; EHNS, European Head and Neck Society; ESMO, European Society for Medical Oncology; ESTRO, European Society for Radiotherapy and Oncology. | |
| **SOURCE OF INFORMATION:** Clinical records. | |
| **CALCULATION PERIOD**: The last 12 months. | |
| **ACCEPTABLE LEVEL: ≥90%.** | |
| **REFERENCES:**   - Machiels JP, René Leemans C, Golusinkiw W, Grau C, Liatra L and Gregorie V on behalf of the EHNS Executive Board, ESMO Guidelines Committee and ESTRO Executive Board. Squamous cell carcinoma of the oral cavity, larynx, oropharynx and hypopharynx: ENS-ESMO-ESTRO Clinical Practice Guidelines for diagnosis, treatment and follow-up. Annals of Oncology. 2020 Nov;31(11):1462-1475. doi: 10.1016/j.annonc.2020.07.011. - Quon H, Vapiwala N, Forastiere A, Kennedy EB, Adelstein DJ et al. Radiation Therapy for Oropharyngeal Squamous Cell Carcinoma: American Society of Clinical Oncology Endorsement of the American Society for Radiation Oncology Evidence-Based Clinical Practice Guideline. Journal Clinic Oncology 2017. 35(36) :4078-4090. doi: 10.1200/JCO.2017.73.8633 - Gourin CG, Herbert RJ, Fahkry C, Quon H, Kang H et al. Quality Indicators of Oropharyngeal Cancer Care in the Elderly. Laryngoscope, 2018; 128:2312–2319. doi: 10.1002/lary.27050. | |

**DIMENSION: RADIOTHERAPY TREATMENT**

| **Criterion 15: Adequacy of therapeutic recommendation** | **PRO-12** |
| --- | --- |
| **INDICATOR: Use of intensity-modulated radiation therapy (IMRT) in radical radiotherapy.** | |
| **DEFINITION:** Appropriate use of IMRT in patients with head and neck cancer undergoing radical radiotherapy. | |
| **FORMULA:** Number of patients with locoregional head and neck cancer with indication for radical radiotherapy receiving IMRT x 100 / Total number of patients with locoregional head and neck cancer for whom radical radiotherapy is indicated. | |
| **INDICATOR of:** Process | |
| **RATIONALE / EXCLUSIONS / CLARIFICATIONS:**  RATIONALE:  IMRT has been shown to reduce long-term toxicity to the oropharynx, nasal cavity, paranasal sinuses, salivary glands, and nasopharynx by delivering a lower radiation dose to the salivary glands, temporal lobe, and auditory (including cochlea) and ocular structures.  EXCLUSIONS:   - Patients who die before starting treatment.   CLARIFICATIONS:  If IMRT is not available, patients should be referred to a centre where IMRT can be conducted.  In the case of stage I or II laryngeal carcinoma, 3D external beam radiation therapy may be used instead of IMRT.  IMRT, intensity-modulated radiation therapy. | |
| **SOURCE OF INFORMATION:** Clinical records. | |
| **CALCULATION PERIOD**: The last 12 months. | |
| **ACCEPTABLE LEVEL: ≥95%.** | |
| **REFERENCES:**   - Machiels JP, René Leemans C, Golusinkiw W, Grau C, Liatra L and Gregorie V on behalf of the EHNS Executive Board, ESMO Guidelines Committee and ESTRO Executive Board. Squamous cell carcinoma of the oral cavity, larynx, oropharynx, and hypopharynx: ENS-ESMO-ESTRO Clinical Practice Guidelines for diagnosis, treatment and follow-up. Annals of Oncology. 2020 Nov;31(11):1462-1475. doi: 10.1016/j.annonc.2020.07.011. - NCCN Clinical Practice Guidelines in Oncology (NCCN Guidelines®). Head and Neck Cancers V3.2021. - NHS Scottish Cancer Taskforce National Cancer Quality Steering Group Final Head and Neck Cancer QPI Document v3.0 and v 4.0– (13^th^ July 2018). - NHS Scottish Cancer Taskforce National Cancer Quality Steering Group (Information Services Division). Head & Neck Cancer Quality Performance Indicators. An Official Statistics publication for Scotland. Patients diagnosed between April 2015 and March 2018. May 2018. | |

**DIMENSION: TREATMENT OF PATIENTS WITH EARLY HEAD AND NECK CANCER**

| **Criterion 16: Adequacy of the therapeutic recommendation** | **PRO-13** |
| --- | --- |
| **INDICATOR: Adequate use of monotherapy in early stages of disease.** | |
| **DEFINITION:** Appropriate indication for monotherapy (surgery or radiotherapy) in patients with early-stage (I and II) head and neck squamous cell cancer. | |
| **FORMULA:** Number of patients with early-stage head and neck squamous cell carcinoma treated with monotherapy (surgery or radiotherapy) x 100 / Total number of patients with early-stage head and neck squamous cell carcinoma for whom treatment is indicated. | |
| **INDICATOR of:** Process | |
| **RATIONALE / EXCLUSIONS / CLARIFICATIONS:**  RATIONALE:  Monotherapy with conservative surgery or radiotherapy (external beam radiotherapy or brachytherapy) is the treatment of choice for patients with squamous cell cancer located in the oropharynx, oral cavity, or vocal cords. Both options provide similar locoregional disease control. Treatment will be selected on an individual basis, taking into consideration greater functionality, less morbidity, patient preference, and experience at the centre where treatment will be delivered.  EXCLUSIONS:   - Patients who die before initiating the first treatment. - Presence of risk factors in the pathological anatomy results that, when assessed by a committee, justify the indication for complementary treatment. | |
| **SOURCE OF INFORMATION:** Clinical records. | |
| **CALCULATION PERIOD**: The last 12 months. | |
| **ACCEPTABLE LEVEL: ≥90%.** | |
| **REFERENCES:**   - Leroy R, De Gent C, Stordeur S, Silvermit G, Verleye L et al. Quality Indicators for the management of Head and Neck Squamous Cell Carcinoma. Belgium Health Care Knowledge 2019 Report. - Machiels JP, René Leemans C, Golusinkiw W, Grau C, Liatra L and Gregorie V on behalf of the EHNS Executive Board, ESMO Guidelines Committee and ESTRO Executive Board. Squamous cell carcinoma of the oral cavity, larynx, oropharynx, and hypopharynx: ENS-ESMO-ESTRO Clinical Practice Guidelines for diagnosis, treatment and follow-up. Annals of Oncology. 2020 Nov;31(11):1462-1475. doi: 10.1016/j.annonc.2020.07.011. - NCCN Clinical Practice Guidelines in Oncology (NCCN Guidelines®). Head and Neck Cancers V3.2021. | |

**DIMENSION: TREATMENT OF PATIENTS WITH HEAD AND NECK CANCER STAGES III and IV**

| **Criterion 17: Adequacy of therapeutic recommendation** | **PRO-14** |
| --- | --- |
| **INDICATOR: Adjuvant chemoradiotherapy with cisplatin in patients with stage III or IV head and neck squamous cell carcinoma.** | |
| **DEFINITION:** Chemoradiotherapy with cisplatin is the adjuvant treatment of choice for patients with stage III and IV squamous cell carcinoma of the oral cavity, larynx, or pharynx with extracapsular spread and/or involved margins (<1 mm) of the primary tumour. | |
| **FORMULA:** Number of patients with stage III and IV head and neck squamous cell carcinoma of the oral cavity, larynx, or pharynx with extracapsular spread and/or involved margins (<1 mm) of the primary tumour receiving chemoradiation with cisplatin x 100 / Total patients with stage III and IV head and neck squamous cell carcinoma of the oral cavity, larynx, or pharynx with extracapsular spread and/or involved margins (<1 mm) of the primary tumour | |
| **INDICATOR of:** Process | |
| **RATIONALE / EXCLUSIONS / CLARIFICATIONS:**  RATIONALE:  The use of systemic treatment should be individualised according to patient characteristics. The chemoradiotherapy (CRT) of choice for patients with locally advanced disease continues to be the concomitant use of cisplatin and radiotherapy.  CRT achieves better locoregional disease control and overall survival than radiotherapy, regardless of tumour location (oral cavity, pharynx, or larynx).  EXCLUSIONS:   - Patients with contraindications to cisplatin.   CLARIFICATIONS:  CRT with cisplatin will be administered tri-weekly or weekly (reach at least the cumulative administered dose of 200 mg/m^2^ with cisplatin).  CRT, chemoradiotherapy. | |
| **SOURCE OF INFORMATION:** Clinical records. | |
| **CALCULATION PERIOD**: The last 12 months. | |
| **ACCEPTABLE LEVEL: ≥90%.** | |
| **REFERENCES:**   - Machiels JP, René Leemans C, Golusinkiw W, Grau C, Liatra L and Gregorie V on behalf of the EHNS Executive Board, ESMO Guidelines Committee and ESTRO Executive Board. Squamous cell carcinoma of the oral cavity, larynx, oropharynx, and hypopharynx: ENS-ESMO-ESTRO Clinical Practice Guidelines for diagnosis, treatment and follow-up. Annals of Oncology. 2020 Nov;31(11):1462-1475. doi: 10.1016/j.annonc.2020.07.011. - NCCN Clinical Practice Guidelines in Oncology (NCCN Guidelines®). Head and Neck Cancers V3.2021. | |

**DIMENSION: TREATMENT OF PATIENTS WITH LOCALLY ADVANCED DISEASE**

| **Criterion 18: Adequacy of therapeutic recommendation** | **PRO-15** |
| --- | --- |
| **INDICATOR: Adequate indication for chemoradiotherapy (CRT) in patients with locally advanced disease.** | |
| **DEFINITION:** The treatment of choice for patients with head and neck squamous cell carcinoma with locally advanced stages (stage III, IVA and IVB) who are not candidates or who do not elect surgical treatment is radiotherapy concomitant with cisplatin or cetuximab**.** | |
| **FORMULA:** Number of patients with locally advanced (stage III, IVA and IVB) head and neck squamous cell carcinoma who are not candidates for surgical treatment and who are treated with CRT x100 / Total patients with locally advanced (stage III, IVA and IVB) head and neck squamous cell carcinoma who are not candidates for surgical treatment and who initiate treatment. | |
| **INDICATOR of:** Process | |
| **RATIONALE / EXCLUSIONS / CLARIFICATIONS:**  RATIONALE:  The standard treatment for patients with locally advanced (stage III, IVA and IVB) squamous cell cancer who are not candidates for surgical treatment will be concomitant CRT with high-dose cisplatin (100 mg/m^2^) or cetuximab.  EXCLUSIONS:   - Patients who die before starting treatment. - Patients with contraindications to systemic treatment.   CLARIFICATIONS:  Cetuximab is indicated in patients who cannot be treated with cisplatin.  CRT, chemoradiotherapy. | |
| **SOURCE OF INFORMATION:** Clinical records. | |
| **CALCULATION PERIOD**: The last 12 months. | |
| **ACCEPTABLE LEVEL: ≥95%.** | |
| **REFERENCES:**   - Machiels JP, René Leemans C, Golusinkiw W, Grau C, Liatra L and Gregorie V on behalf of the EHNS Executive Board, ESMO Guidelines Committee and ESTRO Executive Board. Squamous cell carcinoma of the oral cavity, larynx, oropharynx, and hypopharynx: ENS-ESMO-ESTRO Clinical Practice Guidelines for diagnosis, treatment and follow-up. Annals of Oncology. 2020 Nov;31(11):1462-1475. doi: 10.1016/j.annonc.2020.07.011. - NCCN Clinical Practice Guidelines in Oncology (NCCN Guidelines®). Head and Neck Cancers V3.2021. | |

**DIMENSION: FOLLOW-UP OF PATIENTS WITH LOCALLY ADVANCED DISEASE**

| **Criterion 19: Adequate follow-up of the patient** | **RES-01** |
| --- | --- |
| **INDICATOR: Assessment of response to chemoradiotherapy (CRT) after completing radical treatment in patients with locally advanced (stage III, IVA and IVB) disease.** | |
| **DEFINITION**: Adequate assessment of the response to CRT in patients with locally advanced (stage III, IVA and IVB) head and neck cancer by imaging (CT or FDG-PET functional imaging) and physical examination (inspection of the oral cavity or nasofibroscopy). | |
| **FORMULA**: Number of patients with locally advanced (stage III, IVA and IVB) head and neck cancer treated with CRT and whose response is fully assessed x100 / Total patients with locally advanced (stage III, IVA and IVB) head and neck cancer treated with CRT. | |
| **INDICATOR of:** Result | |
| **RATIONALE / EXCLUSIONS / CLARIFICATIONS:**  RATIONALE:  Not all patients achieve a complete response; some patients may need complementary treatment. Therefore, response assessment must include physical examination and imaging after completion of radical treatment.  EXCLUSIONS:   - Patients who die during treatment. - Patients who die in the interval until the first response assessment. - Patients lost to follow-up because they changed to a different centre.   CLARIFICATIONS:  Evaluation of response by:   - Physical examination (inspection of the oral cavity or nasofibroscopy). - Imaging (CT or FDG-PET functional imaging)   CRT, chemoradiotherapy; CT, computed tomography; FDG-PET, fluorodeoxyglucose positron emission tomography. | |
| **SOURCE OF INFORMATION**: Clinical records. | |
| **CALCULATION PERIOD**: The last 12 months. | |
| **ACCEPTABLE LEVEL: ≥95%.** | |
| **REFERENCES:**   - NCCN Clinical Practice Guidelines in Oncology (NCCN Guidelines®). Head and Neck Cancers V3.2021. - Machiels JP, René Leemans C, Golusinkiw W, Grau C, Liatra L and Gregorie V on behalf of the EHNS Executive Board, ESMO Guidelines Committee and ESTRO Executive Board. Squamous cell carcinoma of the oral cavity, larynx, oropharynx and hypopharynx: ENS-ESMO-ESTRO Clinical Practice Guidelines for diagnosis, treatment and follow-up. Annals of Oncology. 2020 Nov;31(11):1462-1475. doi: 10.1016/j.annonc.2020.07.011. - Bree R, Wolf GT, Keizer B, Nixon I, Hartl D et al. Response assessment after induction chemotherapy for head and neck squamous cell carcinoma: From physical examination to modern imaging techniques and beyond. Head & Neck. 2017; 39:2329–2349. doi: 10.1002/hed.24883. | |

**DIMENSION: FOLLOW-UP OF PATIENTS WITH LOCALLY ADVANCED DISEASE**

| **Criterion 20: Adequate follow-up of the patient** | **RES-02** |
| --- | --- |
| **INDICATOR: Assessment of response to chemoradiotherapy in locally advanced patients (stage III, IVA and IVB) 8–12 weeks after radical treatment completion.** | |
| **DEFINITION**: Adequate assessment of the response to CRT in patients with locally advanced (stage III, IVA and IVB) head and neck cancer by imaging and physical examination 8–12 weeks after treatment completion. | |
| **FORMULA**: Number of patients with locally advanced (stage III, IVA and IVB) head and neck cancer treated with CRT and whose response is fully assessed 8–12 weeks after completing treatment x 100 / Total patients with locally advanced (stage III, IVA and IVB) head and neck cancer treated with CRT. | |
| **INDICATOR of:** Result | |
| **RATIONALE / EXCLUSIONS / CLARIFICATIONS:**  RATIONALE:  Complete morphologic and/or metabolic response may take several months in some head and neck cancers. Additionally, acute radiotherapy-induced inflammation may lead to false positive results of response or equivocal findings of metabolic activity if evaluated too early. Based on this, imaging and physical examination must be delayed 8­–12 weeks after completion of CRT.  EXCLUSIONS:   - Patients for whom tumour progression is suspected before 8–12 weeks. - Patients lost to follow-up because they changed to a different centre.   CLARIFICATIONS:  Evaluation of response by:   - Physical examination (inspection of the oral cavity or nasofibroscopy). - Imaging (CT or FDG-PET functional imaging)   CRT, chemoradiotherapy; CT, computed tomography; FDG-PET, fluorodeoxyglucose positron emission tomography. | |
| **SOURCE OF INFORMATION**: Clinical records. | |
| **CALCULATION PERIOD**: The last 12 months. | |
| **ACCEPTABLE LEVEL**: ≥90%. | |
| **REFERENCES:**   - NCCN Clinical Practice Guidelines in Oncology (NCCN Guidelines®). Head and Neck Cancers V3.2021. - Machiels JP, René Leemans C, Golusinkiw W, Grau C, Liatra L and Gregorie V on behalf of the EHNS Executive Board, ESMO Guidelines Committee and ESTRO Executive Board. Squamous cell carcinoma of the oral cavity, larynx, oropharynx and hypopharynx: ENS-ESMO-ESTRO Clinical Practice Guidelines for diagnosis, treatment and follow-up. Annals of Oncology. 2020 Nov;31(11):1462-1475. doi: 10.1016/j.annonc.2020.07.011. | |

**DIMENSION: ACTIONS TO BE TAKEN BEFORE STARTING TREATMENT**

| **Criterion 21: Appropriate use of diagnostic tests in recurrent and/or metastatic head and neck cancer** | **PRO-16** |
| --- | --- |
| **INDICATOR: Determination of PD-L1 expression.** | |
| **DEFINITION**: PD-L1 expression should be determined in patients with recurrent and/or metastatic head and neck cancer using the Combined Positive Score (CPS). | |
| **FORMULA**: Number of patients with first-line recurrent and/or metastatic stage head and neck cancer in whom PD-L1 expression is determined by CPS x 100 / Total patients with recurrent and/or metastatic stage head and neck cancer. | |
| **INDICATOR** of: Process | |
| **RATIONALE / EXCLUSIONS / CLARIFICATIONS:**  RATIONALE:  Immunotherapy with PD-L1 immune checkpoint inhibitors is a therapeutic strategy that should be considered in patients with recurrent, unresectable and/or metastatic head and neck squamous cell carcinoma for whom surgery and radiotherapy are not valid therapeutic options. To determine the appropriateness of immunotherapy, PD-L1 expression levels in the tumour must be evaluated.  EXCLUSIONS:   - Platinum-refractory patients (progression within 6 months of completing curative treatment). - Patients who are not candidates for immunotherapy. - Patients with no or insufficient tumour specimen for anatomopathological evaluation.   CLARIFICATIONS:  Patients with indication for first line treatment with pembrolizumab (with or without chemotherapy) determined by CPS.  CPS, combined positive score; PD-L1, programmed cell death ligand 1. | |
| **SOURCE OF INFORMATION:** Clinical records. | |
| **CALCULATION PERIOD**: The last 12 months. | |
| **ACCEPTABLE LEVEL: ≥95%.** | |
| **REFERENCES:**   - NCCN Clinical Practice Guidelines in Oncology (NCCN Guidelines®). Head and Neck Cancers V3.2021. - Machiels JP, René Leemans C, Golusinkiw W, Grau C, Liatra L and Gregorie V on behalf of the EHNS Executive Board, ESMO Guidelines Committee and ESTRO Executive Board. Squamous cell carcinoma of the oral cavity, larynx, oropharynx and hypopharynx: ENS-ESMO-ESTRO Clinical Practice Guidelines for diagnosis, treatment and follow-up. Annals of Oncology. 2020 Nov;31(11):1462-1475. doi: 10.1016/j.annonc.2020.07.011. - Burtness B, Harrington K, Greil R et al. pembrolizumab alone or with chemotherapy versus cetuximab with chemotherapy for recurrent or metastatic squamous cell carcinoma of the Head and Neck (KEYNOTE-048): a randomized, open-label, phase-III study. Lancet. 2019. doi: 10.1016/S0140- 6736(19)32591-7 | |

**DIMENSION: TREATMENT OF PATIENTS IN RELAPSED AND/OR METASTATIC STAGE**

| **Criterion 22: Adequate access to approved novel therapies** | **PRO-17** |
| --- | --- |
| **INDICATOR: Access to immunotherapy by eligible patients with recurrent and/or metastatic disease.** | |
| **DEFINITION:** Patients with recurrent and/or metastatic head and neck cancer who meet the appropriate criteria should have access to immunotherapy. | |
| **FORMULA**: Number of patients with recurrent and/or metastatic head and neck cancer who meet criteria to receive immunotherapy x 100 / Total number of patients with recurrent and/or metastatic head and neck cancer who meet criteria to receive immunotherapy. | |
| **INDICATOR of:** Process | |
| **RATIONALE / EXCLUSIONS / CLARIFICATIONS:**  RATIONALE:  Patients with recurrent and/or metastatic head and neck cancer who meet criteria for receiving immunotherapy and have a good risk–benefit profile determined by scientific evidence should have access to available treatments.  EXCLUSIONS:   - Patients who refuse treatment with immunotherapy. - Patients with contraindications for immunotherapy treatment.   CLARIFICATIONS:  Indications for treatment with immunotherapy:   - Nivolumab: platinum-refractory patient (used in radical treatment or in case of recurrent and/or metastatic tumour). - Pembrolizumab: patients who are platinum-sensitive in the first-line (as monotherapy or combined with chemotherapy) and with recurrent and/or metastatic disease, and with expression at PD-L1 expression ≥1 evaluated by CPS.   CPS, combined positive score. | |
| **SOURCE OF INFORMATION**: Clinical records. | |
| **CALCULATION PERIOD**: The last 12 months. | |
| **ACCEPTABLE LEVEL: ≥95%.** | |
| **REFERENCES:**   - NCCN Clinical Practice Guidelines in Oncology (NCCN Guidelines®). Head and Neck Cancers V3.2021. - Machiels JP, René Leemans C, Golusinkiw W, Grau C, Liatra L and Gregorie V on behalf of the EHNS Executive Board, ESMO Guidelines Committee and ESTRO Executive Board. Squamous cell carcinoma of the oral cavity, larynx, oropharynx and hypopharynx: ENS-ESMO-ESTRO Clinical Practice Guidelines for diagnosis, treatment and follow-up. Annals of Oncology. 2020 Nov;31(11):1462-1475. doi: 10.1016/j.annonc.2020.07.011. - Nabil F. Saba, George Blumenschein, Joel Guigay, Lisa Licitra, Jerome Fayette, Kevin J. Harrington, Naomi Kiyota, Maura L. Gillison, Robert L. Ferris, Vijayvel Jayaprakash, Li Li, Peter Brossart. Nivolumab versus investigator’s choice in patients with recurrent or metastatic squamous cell carcinoma of the head and neck: Efficacy and safety in CheckMate 141 by age, Oral Oncology. Volume 96, 2019, Pages 7-14, ISSN 1368-8375. doi: 10.1016/j.oraloncology.2019.06.017. - Harrington KJ, Ferris RL, Blumenschein G Jr, Colevas AD, Fayette J, Licitra L, Kasper S, Even C, Vokes EE, Worden F, Saba NF, Kiyota N, Haddad R, Tahara M, Grünwald V, Shaw JW, Monga M, Lynch M, Taylor F, DeRosa M, Morrissey L, Cocks K, Gillison ML, Guigay J. Nivolumab versus standard, single-agent therapy of investigator's choice in recurrent or metastatic squamous cell carcinoma of the head and neck (CheckMate 141): health-related quality-of-life results from a randomised, phase 3 trial. Lancet Oncol. 2017 Aug;18(8):1104-1115. doi: 10.1016/S1470-2045(17)30421-7. - Burtness B, Harrington KJ, Greil R, Soulières D, Tahara M, de Castro G Jr, Psyrri A, Basté N, Neupane P, Bratland Å, Fuereder T, Hughes BGM, Mesía R, Ngamphaiboon N, Rordorf T, Wan Ishak WZ, Hong RL, González Mendoza R, Roy A, Zhang Y, Gumuscu B, Cheng JD, Jin F, Rischin D; KEYNOTE-048 Investigators. Pembrolizumab alone or with chemotherapy versus cetuximab with chemotherapy for recurrent or metastatic squamous cell carcinoma of the head and neck (KEYNOTE-048): a randomised, open-label, phase 3 study. Lancet. 2019 Nov 23;394(10212):1915-1928. doi: 10.1016/S0140-6736(19)32591-7. | |

**DIMENSION:** **FOLLOW-UP OF PATIENTS IN RECURRENT AND/OR METASTATIC STAGE**

| **Criterion 23: Adequate patient follow-up** | **PRO-18** |
| --- | --- |
| **INDICATOR:** **Tumour Board assessment of treatment of patients with local or systemic recurrence.** | |
| **DEFINITION**: Assessment of patients with local or systemic recurrence by the Tumour Board to consider salvage surgery and/or radiotherapy (including re-irradiation). | |
| **FORMULA**: Number of patients with head and neck cancer with local or systemic recurrence assessed by the Tumour Board x 100 / Total patients with relapsed and/or metastatic head and neck cancer with local or systemic recurrence. | |
| **INDICATOR of:** Process | |
| **RATIONALE / EXCLUSIONS / CLARIFICATIONS:**  RATIONALE:  Coordination between the different specialists involved in the treatment of head and neck cancer is necessary in order to determine the initial therapeutic strategy. Patients should be followed up and, if recurrence is suspected or there is risk for recurrence, the Tumour Board should determine the salvage therapy to use, either surgery or radiotherapy, depending on the patient’s characteristics and prior therapy.  EXCLUSIONS:  Patients who die before they can be evaluated by the Tumour Board.  Patients lost to follow-up because they changed to a different centre.  Patients in need of urgent treatment. | |
| **SOURCE OF INFORMATION**: Clinical records. | |
| **CALCULATION PERIOD**: The last 12 months. | |
| **ACCEPTABLE LEVEL**: ≥90%. | |
| **REFERENCES:**   - NCCN Clinical Practice Guidelines in Oncology (NCCN Guidelines®). Head and Neck Cancers V3.2021. - Leroy R, De Gent C, Stordeur S, Silvermit G, Verleye L et al. Quality Indicators for the management of Head and Neck Squamous Cell Carcinoma. Belgium Health Care Knowledge 2019 Report. - Van Overveld LFJ, Braspenning JCC, Hermens RPMG. Quality indicators of integrated care for patients with head and neck cancer. Clin. Otolaryngol. 2017, 42, 322–329. doi: 10.1111/coa.12724. - Takes RP, Halmos GB, Ridge JA, Bossi P, Merkx MAW, Rinaldo A, Sanabria A, Smeele LE, Mäkitie AA, Ferlito A. Value and Quality of Care in Head and Neck Oncology. Current Oncology Reports (2020) 22: 92. doi: 10.1007/s11912-020-00952-5. | |

**DIMENSION: TREATMENT OF PATIENTS IN RELAPSED AND/OR METASTATIC STAGE**

| **Criterion 24: Adequate therapeutic recommendation** | **PRO-19** |
| --- | --- |
| **INDICATOR: Assessment of second- or third-line therapy in patients with recurrent and/or metastatic head and neck cancer.** | |
| **DEFINITION**: Appropriate indication for second- or third-line treatment in patients with recurrent and/or metastatic head and neck cancer after progression to the previous line of therapy. | |
| **FORMULA:** Number of patients with recurrent and/or metastatic head and neck cancer who are offered second- or third-line treatment due to failure of previous lines of therapy x100 / Total patients with recurrent and/or metastatic head and neck cancer with failure to previous line of therapy. | |
| **INDICATOR of:** Process | |
| **RATIONALE / EXCLUSIONS / CLARIFICATIONS:**  RATIONALE:  Immunotherapy alone or combined with chemotherapy enables use of two lines of therapy with proven efficacy in relapsed and/or metastatic disease.  EXCLUSIONS:   - Patients with advanced head and neck cancer for whom treatment has not been effective and who die before being able to receive new lines of treatment or for whom subsequent lines are not indicated because of the patient’s performance status. - Patients who refuse treatment. - Patients lost to follow-up because they changed to a different centre.   CLARIFICATIONS:  Failure to previous line of therapy is determined by presence of progression or recurrence after treatment. | |
| **SOURCE OF INFORMATION**: Clinical records. | |
| **CALCULATION PERIOD**: The last 12 months. | |
| **ACCEPTABLE LEVEL**: ≥90%. | |
| **REFERENCES:**   - NCCN Clinical Practice Guidelines in Oncology (NCCN Guidelines®). Head and Neck Cancers V3.2021. - Machiels JP, René Leemans C, Golusinkiw W, Grau C, Liatra L and Gregorie V on behalf of the EHNS Executive Board, ESMO Guidelines Committee and ESTRO Executive Board. Squamous cell carcinoma of the oral cavity, larynx, oropharynx and hypopharynx: ENS-ESMO-ESTRO Clinical Practice Guidelines for diagnosis, treatment and follow-up. Annals of Oncology. 2020 Nov;31(11):1462-1475. doi: 10.1016/j.annonc.2020.07.011. | |

**DIMENSION: INCLUSION OF PATIENTS IN CLINICAL TRIALS**

| **Criterion 25: Access to clinical trials** | **PRO-20** |
| --- | --- |
| **INDICATOR: Propose participation in clinical trials** | |
| **DEFINITION:** All patients with head and neck cancer should be considered for participation in available clinical trials that fit their clinical characteristics and therapeutic needs. | |
| **FORMULA:** Number of patients with head and neck cancer participating in clinical trials x 100 / Total number of patients with head and neck cancer seen in the department. | |
| **INDICATOR of:** Process | |
| **RATIONALE / EXCLUSIONS / CLARIFICATIONS:**  RATIONALE:  All patients with head and neck cancer, should be considered for participation in available clinical trials that match their clinical characteristics and therapeutic needs.  CLARIFICATIONS:  Participation in clinical trials should be facilitated to patients if these represent a good therapeutic option, regardless of the hospital in which they take place. In the event that the clinical trial is conducted at another hospital, mediation between centres/departments should be managed. | |
| **SOURCE OF INFORMATION**: Clinical records. | |
| **CALCULATION PERIOD:** The last 12 months. | |
| **ACCEPTABLE LEVEL**: ≥10%. | |
| **REFERENCES:**   - NCCN Clinical Practice Guidelines in Oncology (NCCN Guidelines®). Head and Neck Cancers V3.2021. - NHS Scottish Cancer Taskforce National Cancer Quality Steering Group Final Head and Neck Cancer QPI Document v3.0 and v 4.0– (13th July 2018). - NHS Scottish Cancer Taskforce National Cancer Quality Steering Group Information Services Division. Head & Neck Cancer Quality Performance Indicators. An Official Statistics publication for Scotland. Patients diagnosed between April 2015 and March 2018. May 2018. | |

1. **FOLLOW UP**

**DIMENSION: ADEQUATE PATIENT FOLLOW-UP**

| **Criterion 26: Compliance with progress monitoring** | **PRO-21** |
| --- | --- |
| **INDICATOR: Adequate follow-up of patients after treatment completion.** | |
| **DEFINITION**: Adequate follow-up of patients with head and neck cancer after treatment completion. | |
| **FORMULA:**   1. Number of patients with quarterly follow-up visits during the first two years after completing treatment x 100 / Total number of patients who completed treatment in the past ≤2 years. 2. Number of patients with semi-annual follow-up who completed treatment in 2–5 years x 100 / Total number of patients who completed treatment in the past 2–5 years. | |
| **INDICATOR of:** Process | |
| **RATIONALE / EXCLUSIONS / CLARIFICATIONS:**  RATIONALE:  The purpose of follow-up is to detect locoregional and distant recurrences and the appearance of new tumours in other locations, as well as to monitor long-term toxicities resulting from treatments.  EXCLUSIONS:   - Patients who die before starting the first treatment. - Patients included in a palliative care plan. - Patient decision. - Patients who are followed-up at another centre.   CLARIFICATIONS:  Once their treatment has been completed, patients must have regular follow-up visits with any of the specialists who were involved in their treatment:   - Every 3 months: ≤2 years from treatment completion. - Every 6 months: >2 years and ≤5 years from treatment completion.   In follow-up visits, the medical record will be reviewed and a complete physical examination will be performed. Imaging may also be conducted, as recommended by ESMO and NCCN clinical guidelines, depending on the tumour location and the patient's characteristics.  ESMO, European Society for Medical Oncology; NCNN, National Comprehensive Cancer Network. | |
| **SOURCE OF INFORMATION:** Clinical records. | |
| **CALCULATION PERIOD:** The last 12 months. | |
| **ACCEPTABLE LEVEL:**   1. ≥95% (≤ 1 year and ≤2 years since treatment completion). 2. ≥90% (>2 years and ≤5 years since treatment completion). | |
| **REFERENCES:**   - NCCN Clinical Practice Guidelines in Oncology (NCCN Guidelines®). Head and Neck Cancers V3.2021. - Machiels JP, René Leemans C, Golusinkiw W, Grau C, Liatra L and Gregorie V on behalf of the EHNS Executive Board, ESMO Guidelines Committee and ESTRO Executive Board. Squamous cell carcinoma of the oral cavity, larynx, oropharynx and hypopharynx: ENS-ESMO-ESTRO Clinical Practice Guidelines for diagnosis, treatment and follow-up. Annals of Oncology. 2020 Nov;31(11):1462-1475. doi: 10.1016/j.annonc.2020.07.011. | |

FOLLOW UP

**DIMENSION: ADEQUATE PATIENT FOLLOW-UP**

| **Criterion 27: Adequate follow-up of the patient** | **PRO-22** |
| --- | --- |
| **INDICATOR: Assessment of thyroid function after neck irradiation.** | |
| **DEFINITION**: Adequate assessment of thyroid TSH function every 6–12 months in patients who have received radiotherapy in the neck area. | |
| **FORMULA:** Number of patients with head and neck cancer who have received radiotherapy in the neck area and whose thyroid function is assessed every 6–12 months after completion of radiotherapy x 100 / Total number of patients with head and neck cancer who have received radiotherapy in the neck area. | |
| **INDICATOR of:** Process | |
| **RATIONALE / EXCLUSIONS / CLARIFICATIONS:**  RATIONALE:  Radiotherapy to the neck can alter thyroid function, most commonly resulting in a decrease in thyroid function, which can lead to hypothyroidism followed by thyroiditis and thyrotoxicosis.  EXCLUSIONS:   - Patients who continue to be followed up at another centre.   CLARIFICATIONS:  The initial evaluation will be conducted 6 months after completing radiotherapy.  Monitorisation of thyroid function should be maintained periodically (every 6–12 months) for at least the first two years after completion of radiotherapy in all patients who have had neck irradiation.  TSH, thyroid stimulating hormone. | |
| **SOURCE OF INFORMATION:** Clinical records. | |
| **CALCULATION PERIOD**: The last 12 months. | |
| **ACCEPTABLE LEVEL: ≥80%.** | |
| **REFERENCES:**   - NCCN Clinical Practice Guidelines in Oncology (NCCN Guidelines®). Head and Neck Cancers V3.2021. - Machiels JP, René Leemans C, Golusinkiw W, Grau C, Liatra L and Gregorie V on behalf of the EHNS Executive Board, ESMO Guidelines Committee and ESTRO Executive Board. Squamous cell carcinoma of the oral cavity, larynx, oropharynx and hypopharynx: ENS-ESMO-ESTRO Clinical Practice Guidelines for diagnosis, treatment and follow-up. Annals of Oncology. 2020 Nov;31(11):1462-1475. doi: 10.1016/j.annonc.2020.07.011. - Leroy R, De Gent C, Stordeur S, Silvermit G, Verleye L et al. Quality Indicators for the management of Head and Neck Squamous Cell Carcinoma. Belgium Health Care Knowledge 2019 Report. - Lin CL, Wu SY, Huang WT, Feng YH, Yiu CY, Chiang WF, Ho SY, Lin SH. Subsequent thyroid disorders associated with treatment strategy in head and neck cancer patients: a nationwide cohort study. BMC Cancer. 2019 May 16;19(1):461. doi: 10.1186/s12885-019-5697-y. | |

1. **ASSESSMENT OF HEALTH OUTCOMES**

**DIMENSION: HEALTH OUTCOMES OF PATIENTS WITH HEAD AND NECK CANCER**

| **Criterion 28: Mortality after surgical treatment.** | **RES-03** |
| --- | --- |
| **INDICATOR: Mortality of patients with head and neck cancer after surgery.** | |
| **DEFINITION:** Evaluate post-surgical mortality within 30 and/or 90 days of surgery with radical intent. | |
| **FORMULA:**   1. Number of patients with head and neck cancer who die within 30 days of surgery of causes related to post-surgical complications x 100 / Total number of patients with head and neck cancer receiving surgical treatment. 2. Number of patients with head and neck cancer who die within 90 days after surgery of causes related to post-surgical complications x 100 / Total number of patients with head and neck cancer receiving surgical treatment. | |
| **INDICATOR** of: Result | |
| **RATIONALE / EXCLUSIONS / CLARIFICATIONS:**  RATIONALE:  To evaluate the effectiveness and safety of surgery in patients with head and neck cancer, health outcomes must be evaluated to assess whether they are adequate or protocols should be modified.  EXCLUSIONS:   - Patients lost to follow-up because they changed to another centre.   CLARIFICATIONS:  Possible post-surgical complications: bleeding, thrombus and/or infection. | |
| **SOURCE OF INFORMATION:** Clinical records. | |
| **CALCULATION PERIOD**: The last 12 months. | |
| **ACCEPTABLE LEVEL:**   1. 30 days: ≤5%. 2. 90 days: ≤5%. | |
| **REFERENCES:**   - NHS Scottish Cancer Taskforce National Cancer Quality Steering Group Final Head and Neck Cancer QPI Document v3.0 and v 4.0– (13th July 2018). - Shellenberger TD, Rafael Madero-Visbal R and Randal S. Weber RS. Quality Indicators in Head and Neck Operations. Arch Otolaryngol Head and Neck Surg 2011, 11 (137). doi: 10.1001/archoto.2011.177. - Leroy R, De Gent C, Stordeur S, Silvermit G, Verleye L et al. Quality Indicators for the management of Head and Neck Squamous Cell Carcinoma. Belgium Health Care Knowledge 2019 Report. | |

ASSESSMENT OF HEALTH OUTCOMES.

**DIMENSION: HEALTH OUTCOMES OF PATIENTS WITH HEAD AND NECK CANCER**

| **Criterion 29: Mortality after non-surgical treatment with radical intent.** | **RES-04** |
| --- | --- |
| **INDICATOR: Mortality of patients with head and neck cancer who have received non-surgical treatment with radical intent.** | |
| **DEFINITION**: Evaluate the mortality of patients with head and neck cancer within 30 days of non-surgical treatment with radical intent. | |
| **FORMULA:**  Number of patients with head and neck cancer who die from causes related to non-surgical treatment with radical intent within 30 days of its completion x 100 / Total number of patients with head and neck cancer receiving non-surgical treatment with radical intent. | |
| **INDICATOR** of: Result | |
| **RATIONALE / EXCLUSIONS / CLARIFICATIONS**:  RATIONALE:  To evaluate the effectiveness and safety of non-surgical treatments with radical intent performed in patients with head and neck cancer, the health outcomes must be evaluated to assess whether the results are adequate or changes are needed.  EXCLUSIONS:   - Patients lost to follow-up because they changed to another centre. | |
| **SOURCE OF INFORMATION:** Clinical records. | |
| **CALCULATION PERIOD**: The last 12 months. | |
| **ACCEPTABLE LEVEL:** ≤5% | |
| **REFERENCES:**   - NHS Scottish Cancer Taskforce National Cancer Quality Steering Group Final Head and Neck Cancer QPI Document v3.0 and v 4.0– (13th July 2018). - Shellenberger TD, Rafael Madero-Visbal R and Randal S. Weber RS. Quality Indicators in Head and Neck Operations. Arch Otolaryngol Head and Neck Surg 2011, 11 (137). doi: 10.1001/archoto.2011.177. - Leroy R, De Gent C, Stordeur S, Silvermit G, Verleye L et al. Quality Indicators for the management of Head and Neck Squamous Cell Carcinoma. Belgium Health Care Knowledge 2019 Report. | |
